# Transcriptomic analysis of *Rhipicephalus microplus* hemocytes from female ticks infected with *Babesia bovis* or *Babesia bigemina*

**DOI:** 10.1186/s13071-025-06662-w

**Published:** 2025-02-03

**Authors:** Rubikah Vimonish, Janaina Capelli-Peixoto, Wendell Johnson, Lowell Kappmeyer, Perot Saelao, Naomi Taus, Chungwon Chung, Massaro Ueti

**Affiliations:** 1 Program in Vector-Borne Diseases, Department of Veterinary Microbiology and Pathology, College of Veterinary Medicine, Washington State University, Pullman, WA USA; 2 Animal Disease Research Unit, USDA-ARS, Pullman, WA USA; 3 Veterinary Pest Genetic Research Unit, USDA-ARS, Kerrville, TX USA

**Keywords:** *Babesia*, Hemocytes, Proliferation, Apoptosis, Tick immunity, Differential gene expression, RNA-Seq

## Abstract

**Background:**

Tick hemolymph is a sterile fluid that carries nutrients to maintain tick health. The hemolymph creates a hostile environment for invaders including the destruction of microorganisms by its circulating hemocytes. However, *Babesia* parasites escape and disseminate to other organs through the hemolymph to continue their transmission life cycle. Still, it is unknown how tick hemocytes respond to *B. bovis* or *B. bigemina* infection. In this study, we conducted a transcriptomic analysis of hemocytes from female *Rhipicephalus microplus* ticks infected with *Babesia* parasites to understand how gene expression changes during parasite infection.

**Methods:**

During *Babesia* acute infection, female *R. microplus* ticks were fed on bovines to acquire parasites. Engorged females were collected and incubated to develop *Babesia* kinetes in tick hemolymph. The hemolymph was examined to identify ticks that were highly infected with *Babesia* kinetes. Hemocyte cells were collected from replete female ticks infected with *Babesia bovis* or *Babesia bigemina* to perform high-throughput RNA-sequencing (RNA-Seq) analysis.

**Results:**

This study identified major changes in the gene profile of tick hemocytes during *Babesia* infection. The main groups of hemocyte genes that were altered during *Babesia* infection were associated with metabolism, immunity, and cytoskeletal rearrangement. Upregulated genes were mainly involved in defense mechanisms, while downregulated genes were related to cell proliferation and apoptosis. However, the expression of hemocyte genes varied among *Babesia* species’ infections, and it reflected the changes that occurred in the tick’s physiology, including growth, reproduction, and skeletal muscle development.

**Conclusions:**

The differential gene expression of *R. microplus* hemocytes revealed that genes highly regulated upon *Babesia* infection were related to metabolism, tick immunity, cell growth, apoptosis, development, metabolism, and reproduction. Additional research is necessary to further define the genes that exhibited varying expression levels in hemocytes during the infection. The findings of this study will enhance our understanding on how *Babesia* parasites survive in the hostile environment of ticks and perpetuate their transmission cycle, ultimately contributing to the spread of bovine babesiosis.

**Graphical Abstract:**

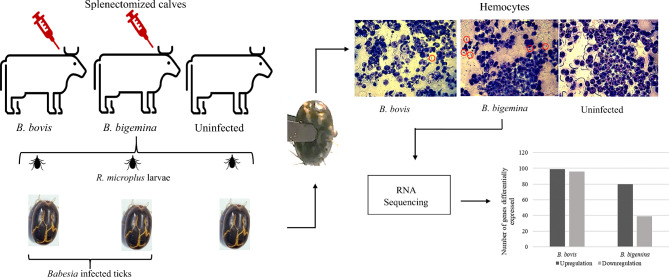

**Supplementary Information:**

The online version contains supplementary material available at 10.1186/s13071-025-06662-w.

## Background

The intraerythrocytic apicomplexan parasites *Babesia bovis* and *Babesia bigemina* are found worldwide [1]. In cattle, babesiosis caused by these *Babesia* parasites leads to significant illness and death, resulting in a substantial economic burden on the livestock industry [2]. *B. bovis* and *B. bigemina* infect cattle through tick transmission. During a tick’s blood meal, red blood cells infected with *Babesia* parasites are ingested and develop into gametes in the tick’s midgut. These gametes fuse to form zygotes, which later develop into motile, elongated kinetes. The kinetes then enter the hemolymph and invade other tick tissues, such as the ovaries. Then, kinetes are transferred to the eggs [3]. However, *Babesia* parasites face a population bottleneck where only a few parasites successfully pass into the next generation of ticks [3, 4]. It remains unclear whether this bottleneck is influenced by the tick’s response mechanisms involving hemocytes.

Circulating hemocytes within a tick’s hemolymph play a crucial role in controlling microbial infections by actively secreting defense molecules, including antimicrobial peptides (AMPs), lysozymes, proteases, protease inhibitors, and lectins [5]. Additionally, hemocytes destroy and control invaders through phagocytosis, encapsulation, nodulation, and coagulation [6]. When a tick encounters microbial invasion from a pathogen such as *Babesia* spp., the number of hemocytes increases to combat the invader [7]. A previous study has shown that *B. bigemina* exhibits motility upon entering the hemolymph and adheres to the membranes of *Rhipicephalus microplus* hemocytes [8]. Despite this knowledge, there is limited information on how *Babesia* infections are managed within the tick hemolymph [9–11]. The lack of in vitro systems for inducing *Babesia* kinete development hinders our understanding of hemocyte responses against *Babesia* parasites. Several studies have employed techniques such as transcriptomics and proteomics in vivo to address these limitations [9–11]. These cutting-edge technologies help us understand the tick-pathogen interactions, specifically, how tick responses change in the presence of pathogens. Understanding the overall response of hemocytes in tick species infected with pathogens such as *Babesia* could greatly aid in comprehending how ticks and pathogens develop strategies to manipulate each other for survival and transmission [12, 13].

By using an RNA-Seq approach, we aimed to investigate two factors: first, the differentially expressed hemocyte genes in response to *Babesia* infection; and second, whether the hemocyte-encoded factors exhibit any species-specific reactions between the two *Babesia* species, *B. bovis* and *B. bigemina*. Our detailed comparative RNA-Seq analysis of *R. microplus* hemocytes identified several differentially expressed genes involved in regulating metabolic pathways, innate immune system pathways, and cytoskeletal rearrangement in response to both *Babesia* species. Analysis of the functional annotation of these differentially expressed hemocyte genes reveals a specific molecular signature in response to *B. bovis* or *B. bigemina* within the hemocyte-encoded factors of its vector tick, *R. microplus*. Notably, the species-specific hemocyte gene response indicates that the integration of tick physiological pathways with innate immune system pathways may play a key role, as hemocyte cell signaling and multicellular processes triggered by invading pathogens support one another. To the best of our knowledge, the current RNA-Seq data provide new insights into how the hemocytes of vector ticks respond to *Babesia* infections and how hemocyte–*Babesia* interactions likely occur during tick innate immune and physiological responses.

## Methods

### Animals, ticks, and pathogens

Animal use in this study was conducted under protocol (IACUC no. 2018-16) as approved by the Institutional Animal Care and Use Committee of the University of Idaho, Moscow, Idaho, USA in accordance with institutional guidelines based on the Animal Welfare Act, the American Society of Animal Science’s Guide for the Care and Use of Agricultural Animals in Research and Teaching, and the US National Institutes of Health Guide for the Care and Use of Laboratory Animals. Four-month-old splenectomized naïve Holstein calves were used to synchronize peak parasitemia with female tick repletion. The La Minita strain of *R. microplus* larvae were applied under a cloth patch on the dorsal region of the animals for the acquisition of *B. bovis* or *B. bigemina*, as previously described [14, 15]. The calves were inoculated with *B. bovis* or *B. bigemina* at around day 14 of postlarval application, when approximately 1% of the ticks were at the molted adult stage. Splenectomized calves (C1, C2, and C3) were infected with ~ 1 × 10^7^ *B. bovis* Texas strain (S74-T3Bo) infected erythrocytes. Splenectomized calves (C4, C5, and C6) were infected with ~ 1 × 10^7^ *B. bigemina* Mexico strain infected erythrocytes. Calves (C7, C8, and C9) were uninfected. Dropped replete female ticks were recovered from all calves, surface sterilized, and incubated at 26 °C and 96% relative humidity to allow for *Babesia* kinete development in tick hemolymph for 8 days, as previously described [3, 16].

### Isolation of tick hemolymph

*Rhipicephalus microplus* engorged female hemolymph samples were collected from three pools of more than 100 ticks heavily infected with *Babesia* kinetes per treatment group: *B. bovis-*infected ticks, *B. bigemina-*infected ticks, and uninfected ticks. The presence of the parasite in the hemolymph was evaluated by removing the distal leg segment of engorged female ticks and exuding hemolymph blotted on a slide for Giemsa staining, as previously described [3]. Engorged female ticks containing more than 50 kinetes per hemolymph smear were selected for hemolymph extraction. Ticks were placed on a sticky tape facing the dorsal side and approximately 200 μl of Hank’s Balanced Salt Solution (Thermo Fisher Scientific, Waltham, MA, USA) was injected into the membrane surrounding the base of the coxal of the fourth leg using a 33-gauge needle (Hamilton Company, Reno, NV, USA), as previously described [14–16]. The cuticle was perforated using a 26-gauge needle and only samples from individual ticks with transparent fluid were collected, pooled, concentrated by centrifugation at 3000 × *g* for 2 min, and suspended using 100 μl of Hank’s Balanced Salt Solution. Giemsa staining was performed to evaluate hemocyte purity. The collected hemocytes were then suspended in TRIzol^®^ (Thermo Fisher Scientific) for DNA and total RNA extractions. Samples were stored at −80 °C until the processing.

### Total RNA extraction

Total RNA from infected and uninfected hemocyte samples was extracted using TRIzol^®^ LS Reagent (ThermoFisher Scientific, Waltham, MA, USA). The hemocytes were centrifuged at 3000 × *g* for 2 min, the pellet suspended in 1 ml of Trizol^®^, and stored at −80 °C. Aqueous and organic phases were separated by adding 0.2 ml of chloroform followed by vigorous shaking, 15 min incubation at room temperature and, centrifugation at 12,000 × *g* for 15 min at 4 °C. The aqueous phase was collected and used for RNA isolation. Five μg of RNase-free glycogen was added to the aqueous phase and the RNA was precipitated using 0.5 ml of 100% isopropanol, followed by 10 min incubation at room temperature and centrifugation at 12,000 × *g* for 10 min at 4 °C. The RNA pellet was washed with 1 ml of 75% ethanol, followed by vortex and centrifugation at 7500 × *g* for 5 min at 4 °C. The RNA pellet was air dried and suspended in RNase-free water and incubated at 60 °C for 10 min. Isolated RNA was treated with TURBO DNase according to the manufacturer’s instructions (Invitrogen, Carlsbad, CA) to eliminate genomic DNA. The concentration of total RNA was measured by Nanodrop spectrophotometer ND 1000 (ThermoFisher Scientific) and tested for residual DNA by polymerase chain reaction (PCR) targeting *R. microplus* calreticulin (AY395254.1). The amplification condition was 98 °C for 2 min, 30 cycles of 98 °C for 30 s, 60 °C for 30 s, 72 °C for 30 s, and final extension at 72 °C for 5 min. The primer sequences used in the PCR analysis are available in Additional File 1. RNA quality was assessed by Nanodrop absorbances at 260/280 nm and 260/230 nm using an Agilent Bioanalyzer Nano RNA chip (Agilent Technologies, Santa Clara, CA). The RNA integrity number values for hemocytes of *B. bovis*-infected ticks were 7.2, 6.1, and 6.8; hemocytes of *B. bigemina*-infected ticks were 8.5, 7.9, and 8.8; and hemocytes of uninfected infected ticks were 8.2, 8.7, and 9.7. Purified RNA samples were stored at −80 °C.

### DNA extraction

Total genomic DNA from infected and uninfected calves (C1 to C9), and the hemocyte samples were extracted using Trizol^®^ Reagent (ThermoFisher Scientific). Defibrinated blood was centrifuged at 800 × *g* for 10 min at 4 °C. The white blood cells were removed, and the blood was washed with Puck’s Saline G (ThermoFisher Scientific). Whole blood (1 ml) was suspended in 9 ml of TRIzol^®^ (ThermoFisher Scientific) and stored at −80 °C. Aqueous and organic phases were separated by following the protocol described above for total RNA extraction and the organic phenol–chloroform phase was used for DNA isolation. The DNA pellet was precipitated using 0.3 ml of 100% ethanol followed by several inversions, 5 min incubation, and centrifugation at 2000 × *g* for 5 min at 4 °C. The DNA pellet was washed twice with 1 ml of 0.01 M sodium citrate/ethanol solution followed by 30 min incubation at room temperature and centrifugation at 2000 × *g* for 5 min at 4 °C. Then, the DNA pellet was washed with 1.5 ml of 75% ethanol followed by 20 min incubation at room temperature and centrifugation at 2000 × *g* for 5 min at 4 °C. The DNA pellet was air dried, suspended in 8 mM NaOH at a concentration of 0.2–0.3 μg/μl, and centrifuged at 12,000 × *g* for 10 min at 4 °C. The supernatant containing the DNA was transferred to a new tube, adding ethylenediaminetetraacetic acid (EDTA) to 1 mM and storing at −20 °C.

### Quantification of *Babesia* DNA

Quantification of *Babesia* infection during tick feeding was performed using primers targeting kinete specific protein (KSP) genes of *B. bovis* (BBOV_I002220) and *B. bigemina* (BBBOND_0206730) genes [14, 15]. Primer sequences and amplicon size are available in Additional File 1. Standard curves were constructed by recombinant KSP of *B. bovis* (BBOV_I002220) and *B. bigemina* (BBBOND_0206730) plasmids. For the molecular cloning, cDNA templates were synthesized by a SuperScript III kit (Invitrogen) from the RNA of *B. bovis* or *B. bigemina* infected hemolymph. PCR reactions targeting KSP of *B. bovis* and *B. bigemina* genes were performed. PCR reactions were performed using 10 µl of REDTaq ReadyMix (Sigma-Aldrich, St. Louis, MO), 500 nM of each primer (Additional Table 1), and 70 ng of template cDNA and RNase/DNase-free water up to 20 µl. Thermocycling conditions consisted of an initial denaturation cycle at 95 °C for 30 s, followed by 35 cycles at 95 °C for 30 s for denaturation, 60 °C for 30 s for annealing, and 72 °C for 1 min for extension, and a final elongation step of 72 °C for 10 min. The amplification products were cloned into the TOPO^®^ 2.1 vector (Invitrogen) following the manufacturer’s guidelines for the TOPO TA Cloning kit. Positive recombinant clones of One shot TOP10 were identified by colony PCR. The recombinant DNAs were obtained using the Wizard® Plus SV Minipreps DNA Purification System (Promega, Madison, WI) and were sequenced in both directions. Serially diluted recombinant plasmid from 10^9^ to 10^0^ copies were used for the standard curves. Quantitative real-time PCR reactions (qPCR) were performed in triplicate for each sample using 10 µl of SsoFast™ EvaGreen^®^ Supermix (BioRad, Hercules, CA), 500 nM of each primer, and 2 µl of template DNA and RNase/DNase-free water up to 20 µl. Thermocycling conditions consisted of an initial denaturation cycle at 95 °C for 30 s, followed by 40 cycles at 95 °C for 5 s for denaturation, 60 °C for 5 s for annealing, and 65 °C for 5 s for extension. The assay was performed in the Biorad CFX real-time PCR detection system (Bio-Rad) and analyzed by qPCR CFX Manager Software (Bio-Rad).

**Table 1:** Tick genes up regulated in response to both *Babesia* infections with a false discovery rate (FDR) < 0.05

Accession no.	Gene symbol	Gene	Fold increase	
			*B. bovis*	*B. bigemina*
XM_037429233.1	LOC119178070	Rm uncharacterized	172.42	825.69
XM_037424754.1	LOC119173969	Rm mitochondrial sodium/calcium exchanger protein-like	146.50	355.42
XM_037413835.1	LOC119161396	Rm antimicrobial peptide microplusin-like	135.75	121.91
XM_037428606.1	LOC119177199	Rm uncharacterized	127.55	710.26
XM_037429480.1	LOC119178287	Rm uncharacterized	117.79	321.71
XM_037419045.1	LOC119167543	Rm gastrula zinc finger protein XICGF8.2DB-like	109.59	67.46
XM_037421222.1	LOC119170156	Rm proline-rich protein HaeIII subfamily 1-like variant X1	107.25	158.24
XM_037414789.1	LOC119162395	Rm 4 kDa defensin-like	103.86	478.62
XM_037433840.1	LOC119184009	Rm uncharacterized	103.47	119.34
XM_037423447.1	LOC119172380	Rm uncharacterized	83.09	339.11
XM_037421224.1	LOC119170156	Rm proline-rich protein HaeIII subfamily 1-like variant X3	81.40	146.87
XM_037429385.1	LOC119178196	Rm Ixodidin-like	79.21	110.6
XM_037413011.1	LOC119160777	Rm putative ribosome biogenesis protein slx9-like	71.24	75.63
XM_037424191.1	LOC119173321	Rm uncharacterized	60.52	21.99
XM_037420540.1	LOC119169498	Rm uncharacterized	57.66	34.47
XM_037414239.1	LOC119161714	Rm uncharacterized	55.61	25.51
XM_037414510.1	LOC119162025	Rm keratin-associated protein 19-2-like	52.32	220.78
XR_005110518.1	LOC119176578	Rm uncharacterized	38.27	18.42
XM_037422643.1	LOC119171798	Rm connectin-like	35.12	44
XR_005109563.1	LOC119169343	Rm uncharacterized	30.28	48.47
XM_037428175.1	LOC119176824	Rm uncharacterized	30.11	12.37
XM_037413526.1	LOC119161172	Rm acanthoscurrin-2-like	29.54	145.12
XM_037420409.1	LOC119169295	Rm uncharacterized	27.30	44.36
XM_037432091.1	LOC119180948	Rm alpha-crystallin A chain-like	27.23	27.93
XM_037418783.1	LOC119167313	Rm papilin-like	27.10	37.81
XM_037429638.1	LOC119178436	Rm uncharacterized	24.07	13.63
XM_037421223.1	LOC119170156	Rm proline-rich protein HaeIII subfamily 1-like	22.02	26.24
XM_037425160.1	LOC119174309	Rm astacin-like metalloprotease toxin 1	21.39	15.21
XR_005108832.1		Rm uncharacterized	17.94	24.28
XM_037416508.1	LOC119164337	atlastin-1-like	17.37	13.94
XM_037413836.1	LOC119161397	antimicrobial peptide microplusin-like	15.09	12.59
XM_037423485.1	LOC119172404	Rm myosin-2 essential light chain-like	13.47	10.65
XM_037423180.1	LOC119172160	Rm serum amyloid A-2 protein-like	12.85	5.34
XM_037422040.1	LOC119170782	Rm uncharacterized	11.62	9.76
XM_037434253.1	LOC119185146	Rm alpha-crystallin B chain-like	11.30	9.94
XM_037413542.1	LOC119161185	Rm transcriptional regulatory protein LGE1-like	10.66	25.18
XM_037416564.1	LOC119164384	Rm acanthoscurrin-1-like	10.62	43.82
XM_037416511.1	LOC119164339	Rm trichohyalin-like	9.95	7.43
XM_037415109.1	LOC119163164	Rm L-threonine 3-dehydrogenase, mitochondrial-like	9.36	9.89
XM_037418012.1	LOC119166691	Rm dipeptidyl peptidase 1-like	9.34	36.13
XM_037425462.1	LOC119174524	Rm B-cell receptor-associated protein 31-like	9.27	13.27
XM_037430262.1	LOC119179189	Rm receptor expression-enhancing protein 5-like	9.00	7.2
XR_005108831.1	LOC119164340	Rm uncharacterized	8.95	6.76
XM_037413530.1	LOC119161174	Rm glycine-rich cell wall structural protein 1-like	8.89	70.05
XM_037422041.1	LOC119170783	Rm uncharacterized	8.12	6.33
XM_037435443.1	LOC119187251	Rm legumain-like	6.46	4.21
XM_037424660.1	LOC119173874	Rm glutathione S-transferase-like	6.00	8.83
XM_037427091.1	LOC119175995	Rm ADP-ribose glycohydrolase ARH3-like	5.91	8.82
XM_037435686.1	LOC119187542	Rm cytochrome C	5.46	6.39
XM_037434254.1	LOC119185148	Rm alpha-crystallin B chain-like	4.78	4.67
XM_037423329.1	LOC119172279	Rm HIG1 domain family member 1A, mitochondrial-like	4.71	5.16
XR_005109227.1	LOC119167468	Rm protein Skeletor	3.69	4.59
XR_005109132.1	LOC119166740	Rm ribonuclease Oy-like	3.64	3.57
XM_037427193.1	LOC119176074	Rm 23 kDa integral membrane protein-like	2.60	2.8

### Constructing RNA-Seq libraries

The RNA-Seq libraries of nine samples (three samples per treatment group) were constructed using the Illumina TruSeq mRNA stranded protocols (Illumina San Diego, CA). Briefly, messenger RNA (mRNA) was enriched from total RNA using poly-A selection and chemically fragmented. The mRNA was synthesized into double-stranded cDNA using reverse transcriptase. Double-stranded cDNA was end-repaired and polyadenylated, and Illumina dual index adapters were ligated to the cDNA samples followed by nine cycles of PCR amplification. The cDNA libraries were quantified, normalized, and multiplexed, as previously described [14]. For the sequencing of cDNA libraries, the multiplexed samples were sequenced on three lanes of a HiSeq 2500 (Illumina), and FASTQ files were generated with pipelines for the removal of adapter sequences from the 3′ ends of reads.

### Differential gene expression

The combined RNA-Seq run resulted in separate FASTQ files representing each library containing sequenced paired reads and quality control data. The raw paired-end reads were quality controlled using Trimmomatic (http://www.usadellab.org/cms/index.php?page=trimmomatic) [17] and checked using FastQC (https://statics.teams.cdn.office.net/evergreen-assets/safelinks/1/atp-safelinks.html) [18]. Low-quality reads were filtered before being *quasi-mapped* using Salmon (version 1.10.1) (https://salmon.readthedocs.io/en/nb/salmon.html) [19] to the *R. microplus* (BIME_Rmic_1.3) gene set. Approximately 45 million paired-end reads were mapped, and differentially expressed genes were identified using DESeq2 (https://bioconductor.org/packages/release/bioc/html/DESeq2.html) [20] by filtering for an independent hypothesis weighting adjusted *p *value of < 0.05. Genes without at least one read per million mapped reads across all three samples within a group were removed, as previously described [14]. The false discovery rate (FDR) method was employed to correct for multiple testing. Significant differential expression of genes was determined by a log fold change (log2 FC) value greater than or equal to ± 1 and the FDR set to < 0.05. Genes were considered upregulated with a log2 FC ≥ 1 or downregulated with a log2 FC ≤ −1. The *p*-values were adjusted for multiple testing according to FDR correction [21]. The FDR of differentially expressed genes and their corresponding *p*-values of this study are provided in Additional File 2. Tick genes regulated in response to *Babesia* infection were searched against the reference genome of *R. microplus* (GCA_013339725.1). Gene ontology (GO) analysis was performed using ShinyGO 0.80 [22] consisting of AmiGO IDs and terms (http://amigo.geneontology.org/amigo/term/), and the heat maps were generated using iDEP 2.01 [23]. All raw and processed RNA sequencing data generated in this study were submitted to the NCBI Gene Expression Omnibus (https://www.ncbi.nlm. nih.gov/geo/) under accession number GSE243493.

### Validation of RNA-Seq data by qRT-PCR

A subset of hemocytes (biological replicates) from female ticks that were fed on the same animals used to generate the cDNA libraries were utilized for qPCR validation. A total of 200 ng of total RNA from hemocytes of *B. bovis*-infected ticks, *B. bigemina*-infected ticks, and uninfected ticks were treated with the TURBO DNA-*free*™ Kit (Invitrogen) and synthesized into cDNA using the SuperScript™ III First-Strand Synthesis System (Invitrogen). The cDNAs were used as templates in qRT-PCR reactions by using SsoFast™ EvaGreen^®^ Supermix (BioRad). The reactions were performed in triplicate for each sample and the reaction mixture consisted of 500 nM of each primer set targeting the gene of interest (Additional File 1), 10 µl of SsoFast EvaGreen Supermix, 25 ng of cDNA, and RNase/DNase-free water up to 20 µl. The qPCR thermal cycling conditions consisted of an initial denaturation cycle at 95 °C for 30 s, followed by 40 cycles at 95 °C for 5 s for denaturation, 60 °C for 5 s for annealing, and 65 °C for 5 s for extension, and the reaction was performed in a CFX96™ real-time PCR detection system (Bio-Rad). CFX Maestro Software 2.3 (Bio-Rad) was used to obtain quantification cycle (Cq) values for the analysis. Primer efficiencies of genes of interest and reference genes were calculated through a 1:10 serial dilution standard curve containing 5 points. Relative gene expression was calculated by using the method that involved geometric averaging of multiple reference genes, as previously described. [24]. Results were collected as the mean relative gene expression of each gene with a standard deviation. An unpaired *t*-test was performed to determine the statistically significant difference in gene expression between groups. Three *R. microplus* genes that were previously shown as suitable internal controls were selected to use as reference genes, including 40S ribosomal protein S3a (XM_037430639.1) [25], ribosomal protein L4 (CV447629.1) [26], and glyceraldehyde-3-phosphate dehydrogenase (CK180824) [26] to normalize transcript levels in the qRT-PCR data. Five differentially expressed genes were validated, including microplusin-like (XM_037413835.1), defensin-like (XM_037414789.1), homeodomain-interacting protein kinase 2-like (XM_037422147.1), protein toll-like (XM_037418714.1), and terminal nucleotidyltransferase 5C-like (XM_037426165.1).

## Results and discussion

### Differential expression of tick hemocyte genes in response to *Babesia* infection

Replete female ticks were collected during the peak of *Babesia* parasitemia. The level of *B. bovis* in the peripheral blood of C1, C2, and C3 during tick feeding was 1 × 10^6.0^, 1 × 10^5.5^, and 1 × 10^5.6^ parasites/ml of blood, respectively. The parasitemia level in the peripheral blood of C4, C5, and C6 infected with *B. bigemina* during tick feeding was 1 × 10^5.8^, 1 × 10^5.4^, and 1 × 10^4.3^ parasites/ml of blood, respectively. The levels of *B. bovis* in the hemolymph from repleted female ticks fed on C1, C2, and C3 were 1 × 10^4.5^, 1 × 10^5.6^, and 1 × 10^4.8^ parasites/sample, respectively. The levels of *B. bigemina* in the hemolymph from repleted female ticks fed on C4, C5, and C6 were 1 × 10^4.6^, 1 × 10^5.7^, and 1 × 10^5.5^ parasites/sample, respectively.

**Fig. 1:**
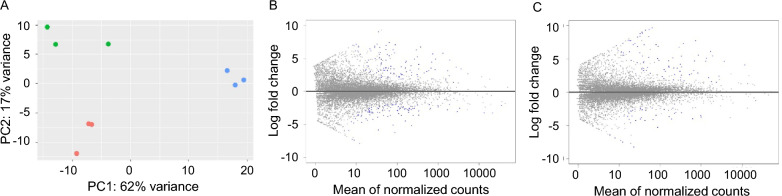
Differential gene expression by *R. microplus* hemocytes in response to *B. bovis* or *B. bigemina* infections. **A** The principal component analysis (PCA) plot between hemocytes from uninfected ticks (blue), hemocytes from ticks infected with *B. bovis* (green), and hemocytes from ticks infected with *B. bigemina* (red). **B** MA (log ratio versus abundance) plot showing the relationship of the log fold-change in gene expression by hemocytes from ticks infected with *B. bovis* versus the mean of normalized counts. **C** MA (log ratio versus abundance) plot showing the relationship of the log fold-change in gene expression by hemocytes from ticks infected with *B. bigemina* versus the mean of normalized counts

Hemocytes of repleted female ticks fed on *Babesia*-infected animals were used to determine differential gene expression in response to *Babesia* kinetes in the hemolymph. The dataset showed differential expression of tick genes by the hemocyte cells in response to *B. bovis* or *B. bigemina* infection. The assembly data of approximately 45 million reads mapped per sample allowed for the identification of tick genes that were upregulated or downregulated in response to infection. Principal component analysis of the RNA-Seq data resulted in separation of the biological replicates of the same condition into three distinct groups including hemocytes from uninfected ticks and hemocytes from ticks infected with either *B. bovis* or *B. bigemina* (Fig. 1A). The first principal component showed 62% of the variation between groups and the second principal component showed that replicates were similar to each other (Fig. 1A). Figure 1 also shows the relationship of the gene fold-change of genes found in the hemocytes infected with *B. bovis* (Fig. 1B) or *B. bigemina* (Fig. 1C) versus the normalized mean expression counts.

**Fig. 2:**
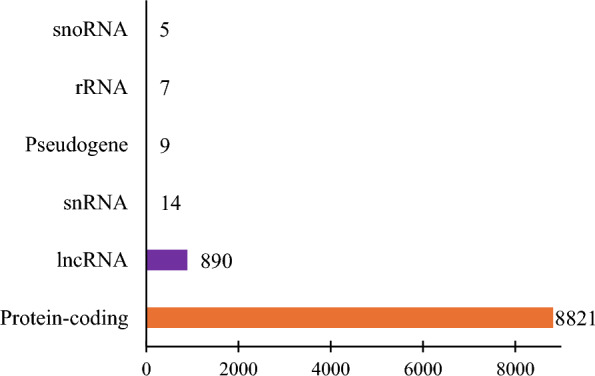
Biotypes of transcripts expressed by *R. microplus* hemocytes. The biotypes were identified using iDEP 2.01 and the classification was based on the Ensembl annotation system [22]

*Rhipicephalus microplus* hemocytes expressed protein-coding genes, long noncoding RNAs (lncRNAs), pseudogenes, ribosomal ribonucleic acids (rRNAs), small nucleolar RNAs (snoRNAs), and small nuclear RNA (snRNAs) (Fig. 2) up to the total number of 11,969 transcripts (Additional File 2).

**Fig. 3:**
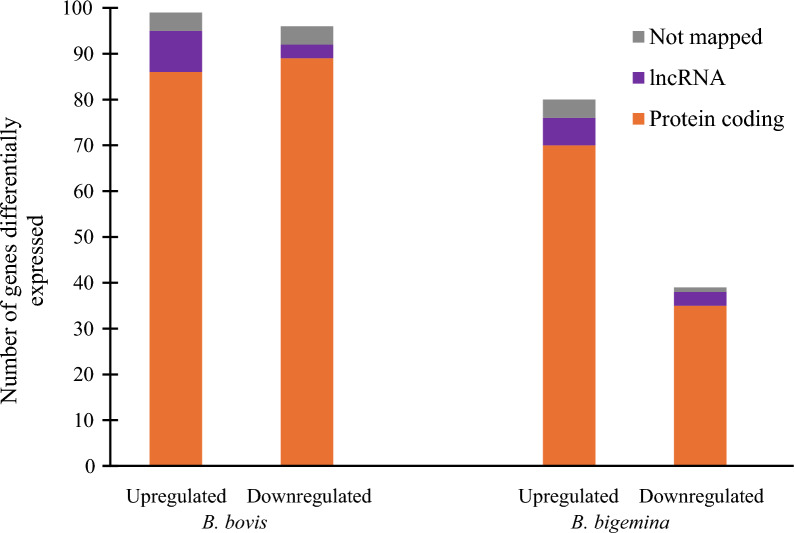
Number of differentially expressed hemocyte transcripts from ticks infected with either *B. bovis* or *B. bigemina*. Upregulated and downregulated tick genes in response to *Babesia* infection with an FDR < 0.05

We identified 175 protein-coding genes, 12 lncRNAs, and 8 genes that were not found by the Ensembl annotation system (not mapped) that were regulated during *B. bovis* infection (Fig. 3). The *B. bigemina* infection included 103 protein-coding genes, 9 lncRNAs, and 5 genes that were not found by the Ensembl annotation system (Fig. 3). The genes that were upregulated and downregulated had an FDR < 0.05. Corresponding FDR and associated *p*-values of differentially expressed genes are provided in Additional File 2. This RNA-Seq study validates and discusses the differentially regulated protein-coding genes that may be critical in controlling the *Babesia* parasite during tick infection. Differentially regulated putative tick lncRNAs were identified in this study; however, it is unknown whether they play any role against tick-borne pathogens or not. Differentially regulated protein coding genes in response to *Babesia* infection as positioned in *R. microplus* chromosomes (21) are shown in Additional File 3.

### Validation of RNA-Seq data by quantitative reverse transcriptase-PCR (qRT-PCR)

**Fig. 4:**
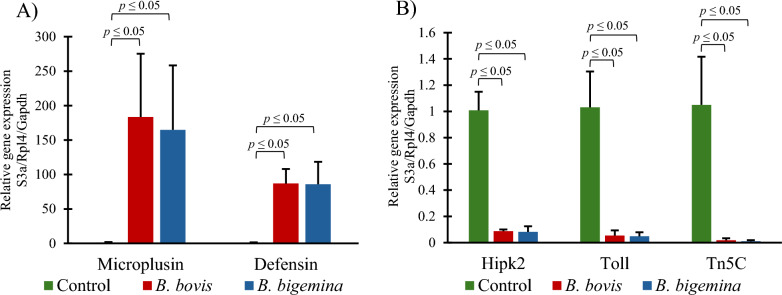
Relative expression of hemocyte genes during *Babesia* infection. **A** Upregulated genes: microplusin-like and defensin-like. **B** Downregulated genes: homeodomain-interacting protein kinase 2-like (Hipk2), protein toll-like (Toll), and terminal nucleotidyltransferase 5C-like (Tn5c)

The mean relative gene expression was compared between hemocytes from infected female ticks with *B. bovis* or *B. bigemina* and uninfected control female ticks. The data obtained using qRT-PCR corroborated results from high-throughput RNA-Seq. The mean relative gene expression of microplusin-like and defensin-like of hemocytes from female ticks infected with *B. bovis* or *B. bigemina* was significantly higher than in the control group (Fig. 4A). The relative gene expression of homeodomain-interacting protein kinase 2-like, protein toll-like, and terminal nucleotidyltransferase 5C-like was significantly lower from ticks infected with *B. bovis* or *B. bigemina* as compared with the uninfected control group (Fig. 4B). Additional genes validated by qRT-PCR are provided in Additional File 4.

Also, 40S ribosomal protein S3a (S3a), ribosomal protein L4 (Rp14), and glyceraldehyde-3-phosphate dehydrogenase (Gapdh) were used for qRT-PCR normalization.

### Hemocyte gene upregulation occurs mutually in *B. bovis *and* B. bigemina* infections

**Fig. 5:**
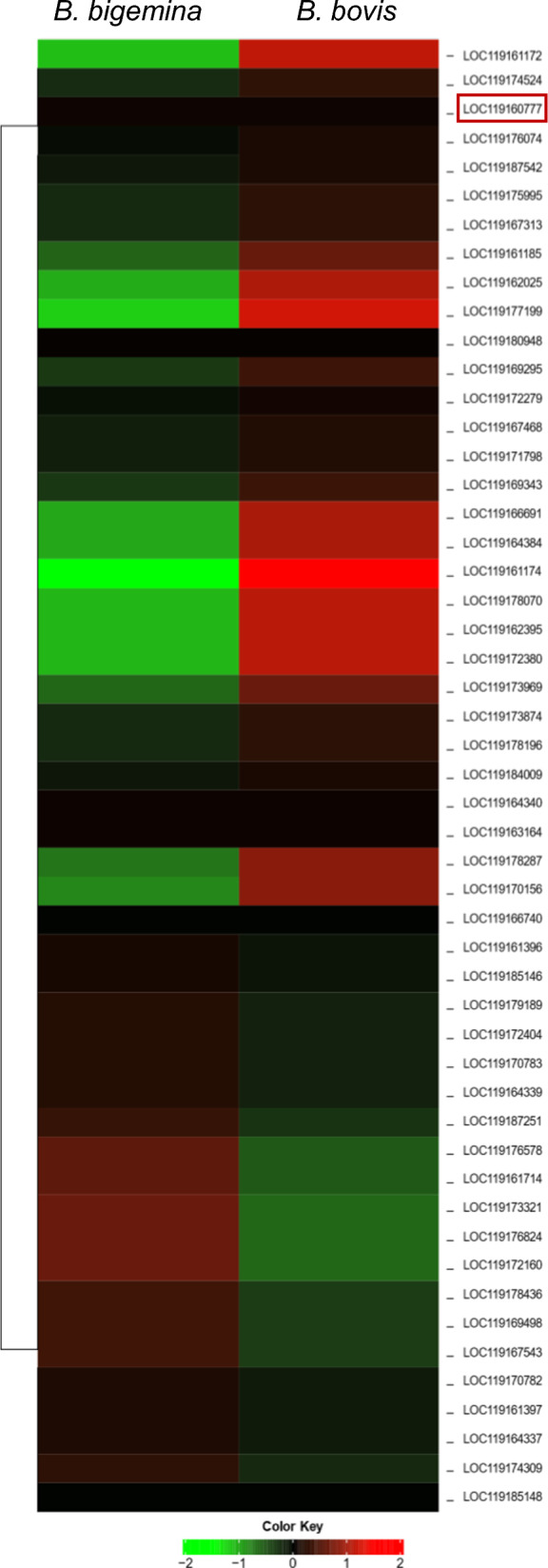
Hierarchical clustering of upregulated hemocyte gene expression following *Babesia* infection. The red frame refers to the putative ribosome biogenesis protein (XM_037413011.1) that regulates cellular metabolism and biogenesis

We found that 54 tick genes were upregulated regardless of the infecting *Babesia* species. Out of 54 upregulated genes, 48 were identified as protein-coding genes, 3 were lncRNAs, and 3 were not found by the Ensemble annotation system [22]. The hierarchical clustering of log fold-change of upregulated hemocyte gene expression suggests tick hemocyte response to *B. bovis* infection is not identical to *B. bigemina* infection (Fig. 5). The upregulation fold range of protein-coding genes was between 2.0-fold and 825.0-fold, and for the putative lncRNAs (XR_005109563.1, XR_005110518.1, and XR_005108831.1) it was between 7.0-fold and 48.0-fold. The fold increase in hemocyte genes was higher in response to *B. bigemina* infection than to *B. bovis* infection (Table 1). Thus, a few genes exhibited comparable expression between both *Babesia* parasites, including the putative ribosome biogenesis protein (XM_037413011.1, highlighted in the heat map), which was expressed similarly during *B. bovis* (71.0-fold) infection and *B. bigemina* (76.0-fold) infection. This gene is involved in regulating cellular metabolism and biogenesis, along with other upregulated genes (Table 2).

**Table 2:** Upregulated hemocyte genes following the *Babesia* infection, grouped by biological process, molecular function, and cellular component categories defined by high-level Gene Ontology (GO) terms

	N	High-level GO category	Genes
Biological process	11	GO:0006807 nitrogen compound metabolic process GO:0071704 organic substance metabolic process	XM_037413011.1 XM_037418012.1 XR_005109132.1 XM_037418783.1 XM_037419045.1 XM_037420540.1 XM_037424660.1 XM_037425160.1 XM_037428175.1 XM_037429385.1 XM_037435443.1
	10	GO:0044238 primary metabolic process	XM_037413011.1 XM_037418012.1 XR_005109132.1 XM_037418783.1 XM_037419045.1 XM_037420540.1 XM_037425160.1 XM_037428175.1 XM_037429385.1 XM_037435443.1
	9	GO:0044237 cellular metabolic process	XM_037413011.1 XR_005109132.1 XM_037418783.1 XM_037419045.1 XM_037420540.1 XM_037424660.1 XM_037428175.1 XM_037429385.1 XM_037435686.1
	4	GO:0050789 regulation of biological process GO:0065007 biological regulation GO:0019222 regulation of metabolic process GO:0050794 regulation of cellular process	XM_037418783.1 XM_037419045.1 XM_037420540.1 XM_037429385.1
	3	GO:0048519 negative regulation of biological process GO:0065009 regulation of molecular function	XM_037418783.1 XM_037420540.1 XM_037429385.1
	2	GO:0051179 localization GO:0051234 establishment of localization	XM_037424754.1 XM_037425462.1
	1	GO:0050896 response to stimulus GO:0006950 response to stress	XM_037414789.1
	1	GO:0071840 cellular component organization or biogenesis GO:0044085 cellular component biogenesis	XM_037413011.1
	1	GO:0009058 biosynthetic process	XM_037419045.1
	1	GO:0033036 macromolecule localization GO:0051641 cellular localization	XM_037425462.1
	1	GO:0044419 biological process involved in interspecies interaction between organisms	XM_037418783.1
Molecular function	7	GO:0016787 hydrolase activity	XM_037416508.1 XM_037418012.1 XR_005109132.1 XM_037425160.1 XM_037427091.1 XM_037428175.1 XM_037435443.1
	7	GO:0043167 ion binding	XM_037416508.1 XM_037423485.1 XM_037425160.1 XM_037428175.1 XM_037432091.1 XM_037434254.1 XM_037435686.1
	4	GO:0005515 protein binding	XM_037416508.1 XM_037422643.1 XM_037424191.1 XM_037424660.1
	3	GO:0016740 transferase activity	XM_037424660.1 XM_037428175.1 XM_037428606.1
	3	GO:0030234 enzyme regulator activity GO:0004857 enzyme inhibitor activity GO:0004866 endopeptidase inhibitor activity GO:0004867 serine-type endopeptidase inhibitor activity GO:0016755 aminoacyltransferase activity GO:0030414 peptidase inhibitor activity GO:0061134 peptidase regulator activity GO:0061135 endopeptidase regulator activity GO:0098772 molecular function regulator activity	XM_037418783.1 XM_037420540.1 XM_037429385.1
	3	GO:0097159 organic cyclic compound binding GO:1,901,363 heterocyclic compound binding	XM_037416508.1 XR_005109132.1 XM_037435686.1
	1	GO:0005215 transporter activity GO:0022857 transmembrane transporter activity	XM_037424754.1
	1	GO:0009055 electron transfer activity GO:0016491 oxidoreductase activity	XM_037435686.1
	1	GO:0003700 DNA-binding transcription factor activity	XM_037419045.1
	1	GO:0016829 lyase activity	XR_005109132.1
	1	GO:0036094 small molecule binding GO:0097367 carbohydrate derivative binding	XM_037416508.1
Cellular component	8	GO:0016020 membrane	XR_005109227.1 XM_037423329.1 XM_037424754.1 XM_037425462.1 XM_037427193.1 XM_037429385.1 XM_037430262.1 XM_037435686.1
	3	GO:0005576 extracellular region	XM_037418783.1 XM_037420540.1 XM_037423180.1
	3	GO:0043226 organelle GO:0043227 membrane-bounded organelle	XM_037413011.1 XM_037425462.1 XM_037435686.1
	2	GO:0031974 membrane-enclosed lumen GO:0043233 organelle lumen	XM_037413011.1 XM_037435686.1
	1	GO:0032991 protein-containing complex GO:1990904 ribonucleoprotein complex GO:0043228 non-membrane-bounded organelle	XM_037413011.1
	1	GO:0005615 extracellular space	XM_037420540.1
	1	GO:0005789 endoplasmic reticulum membrane GO:0012505 endomembrane system GO:0031090 organelle membrane GO:0042175 nuclear outer membrane-endoplasmic reticulum membrane network	XM_037425462.1
	1	GO:0070469 respirasome	XM_037435686.1

N: the number of upregulated hemocyte genes that fall under a similar GO term

This elevated cell metabolism may benefit *Babesia* parasites in that parasites often lack key biosynthetic pathways and rely on their hosts for nutrients. Plasma membrane transporter proteins help parasites to salvage nutrients [27]. Mitochondrial sodium/calcium exchanger protein-like (XM_037424754.1) is a transporter protein found in the *R. microplus* hemocyte membrane (Table 2), and it is upregulated in response to *Babesia* infection (*B. bovis* 146.0-fold and *B. bigemina* 355.0-fold).

**Fig. 6:**
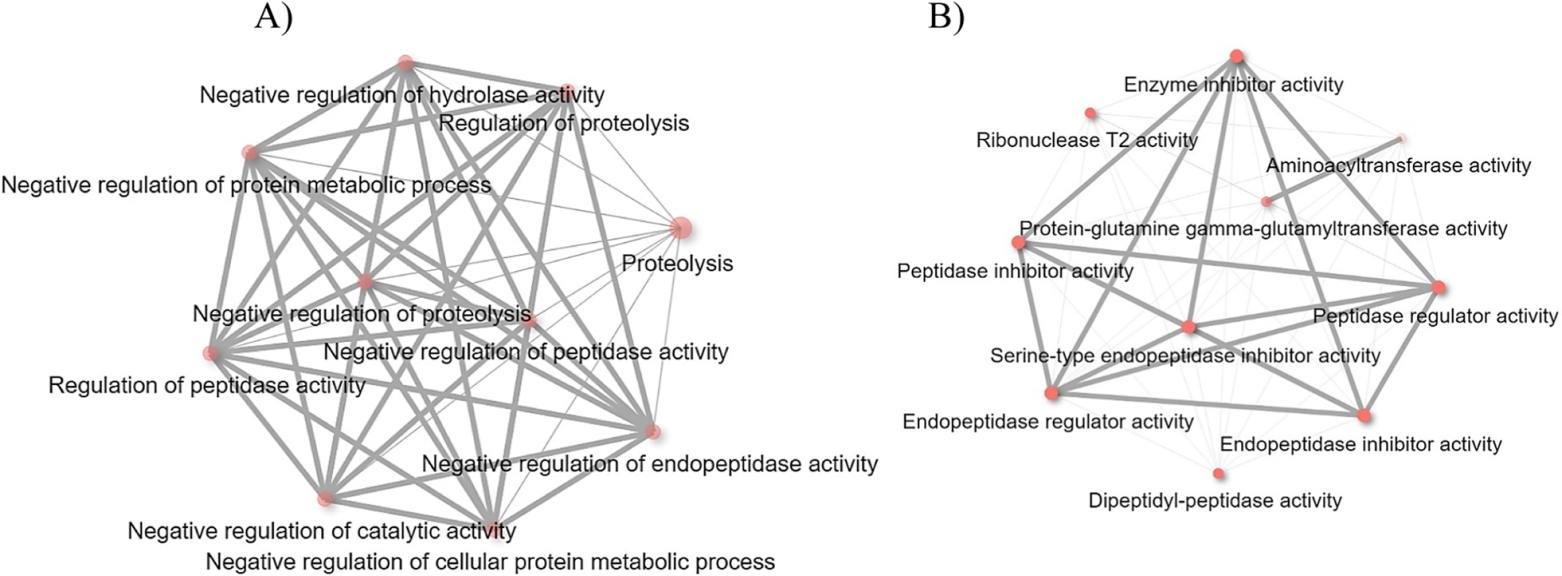
Biological process (**A**) and molecular pathways (**B**) related to upregulated hemocyte genes following *Babesia* infection. Each node of the network represents an enriched GO term and the related GO terms are connected by a line. The thickness of the line reflects the percent of overlapping genes, and the size of the node corresponds to the number of genes

The *Babesia* infection differentially regulated the biological process pathways involved in cellular protein metabolism (Table 2 and Fig. 6A). Upregulated genes, including ixodidin-like (XM_037429385.1), an uncharacterized gene (XM_037420540.1), and papilin-like (XM_037418783.) were identified in the biological process pathways that have the molecular function of serine-type endopeptidase inhibitor or peptidase inhibitor activity (Table 2). Peptidase inhibitors of hard ticks are known to control phagocytosis of microbes by tick hemocytes [28]. Knockdown of the *Ixodes ricinus* pan-protease inhibitor-α2M reduced the phagocytosis of gram-negative bacterium, *Chryseobacterium indologenes*. It was suggested that phagocytosis is mediated by the interaction between α2M and the bacterium’s virulence factor, metalloprotease [29]. Further, α2M of *Dermacentor variabilis* is upregulated in response to rickettsial infection [30], supporting current RNA-Seq findings of elevated protease inhibitor activities of *R. microplus* hemocytes upon *Babesia* infection. *R. microplus* ixodidin, which was identified in this study, is suggested to have inhibitory properties against serine proteases and also shown to be involved in the growth inhibition of *Micrococcus luteus* and *Escherichia coli* [31]. The network of molecular function pathways (Fig. 6B) identified among upregulated hemocyte genes indicated that *R. microplus* hemocytes may have a defense mechanism toward *Babesia* parasites that is composed of peptidase inhibitors.

*Rhipicephalus microplus* hemocytes exhibited a response to *Babesia* infection similar to the stimulus and stress response by 4 kDa defensin-like (XM_037414789.1) with a 104.0-fold increase in response to *B. bovis* and a 479.0-fold increase in response to *B. bigemina* (Table 2). Defensins belong to the largest group of AMPs with a broad spectrum of antimicrobial activity and are part of the innate immune system [32]. Defensins of arthropods destroy microbes by forming pores in their membranes, causing the membrane to become permeable. A previous study indicated that *H. longicornis* defensin can prevent or retard proliferation of *Theileria equi* and *Babesia microti* merozoites [33]. In addition to defensin, other antimicrobial peptide genes were also upregulated in response to *Babesia* infection such as microplusin-like (XM_037413835.1) with a response to *B. bovis* (136.0-fold) and to *B. bigemina* (122.0-fold), acanthoscurrin-2-like (XM_037413526.1) in response to *B. bovis* (29.0-fold) and to *B. bigemina* (145.0-fold), and acanthoscurrin-1-like (XM_037416564.1) in response to *B. bovis* (11.0-fold) and to *B. bigemina* (44.0-fold), suggesting *R. microplus* hemocytes elevate the amount of AMPs or/and regulate its innate immune response against *Babesia*. However, the mode of antiparasitic activity by AMPs on *Babesia* parasites has yet to be determined.

### Hemocyte gene upregulation restricted to *B. bovis* infection

RNA-Seq revealed that 45 tick genes were upregulated in response to *B. bovis* infection but not *B. bigemina* infection. Out of the 45 upregulated genes, 38 were identified as protein-coding genes, 6 were lncRNAs, and 1 was not found by the Ensemble annotation system [22]. Differentially regulated hemocyte genes, upon *B. bovis* infection, showed the upregulation fold ranged between 3.0-fold and 537.0-fold for protein-coding genes, and between 7.0-fold and 49.0-fold for putative lncRNAs (XR_005111542.1, XR_005108161.1, XR_005108162.1, XR_005110086.1, XR_005110507.1, and XR_005108103.1) (Table 3). The presence of *B. bovis* kinetes in the hemolymph drastically elevated the hemocyte gene expression of cytoskeletal components connected to the sarcomere and other components of striated muscle contraction (Table 4). Invertebrate striated muscle contraction machinery comprises sarcomeres formed with thick myosin filaments and thin actin filaments. Contraction and relaxation are regulated through the thin filament-associated troponin complex. The troponin complex has three protein subunits. The calcium receptor subunit (troponin C), the inhibitory subunit (troponin I), and the tropomyosin-binding/thin filament-anchoring subunit (troponin T) [34]. The gene expression of the troponin complex of the *R. microplus* hemocyte was substantially upregulated in response to *B. bovis* infection. *R. microplus* troponin C (XM_037426593.1), troponin 1-like (XM_037421516.1), and troponin T skeletal muscle-like (XM_037421514.1) were upregulated 538.0-fold, 50.0-fold, and 89.0-fold, respectively (Table 3). Along with the troponin complex, *R. microplus* muscle LIM protein 1-like (XM_037420308.1) was differentially regulated (33.0-fold increase) in response to *B. bovis* infection. Muscle LIM protein plays a role in skeletal muscle recovery and maintenance [35].

**Table 3:** Tick genes upregulated in response to *B. bovis* infection only with an FDR < 0.05

Accession no.	Gene	Fold increase
XM_037421516.1	Rm troponin 1-like,	537.61
XM_037428655.1	Rm uncharacterized	144.70
XM_037413134.1	Rm leucine-rich repeat-containing protein 20-like	128.91
XM_037421514.1	Rm troponin T skeletal muscle-like	89.26
XR_005108162.1	Rm uncharacterized	71.43
XM_037435584.1	Rm uncharacterized	71.04
XM_037426593.1	Rm troponin C	50.27
XR_005108161.1	Rm uncharacterized	49.40
XR_005111542.1	Rm uncharacterized	48.89
XM_037422227.1	Rm glycine-rich RNA-binding protein 3, mitochondrial-like	42.86
XM_037420308.1	Rm muscle LIM protein 1-like	33.11
XM_037424720.1	Rm uncharacterized	32.74
XR_005110086.1	Rm uncharacterized	21.76
XM_037422464.1	Rm uncharacterized	20.41
XM_037418910.1	Rm uncharacterized	14.36
XR_005108103.1	Rm uncharacterized	14.09
XM_037414240.1	Rm uncharacterized	12.14
XM_037435231.1	Rm 40S ribosomal protein S17-like	12.11
XM_037418417.1	Rm uncharacterized	12.05
XM_037433338.1	Rm uncharacterized	11.56
XM_037412258.1	Rm uncharacterized	10.87
XM_037418507.1	Rm uncharacterized	10.83
XM_037425790.1	Rm uncharacterized	10.35
XM_037422759.1	Rm uncharacterized	9.40
XM_037416570.1	Rm ribosomal protein rpl-36.A	9.37
XM_037417133.1	Rm complement C3-like	8.44
XM_037427452.1	Rm *A. superbus* venom factor 1-like	8.37
XM_037412516.1	Rm uncharacterized	7.96
XM_037428883.1	Rm snake venom vascular endothelial growth factor toxin cratrin-like	7.69
XM_037427855.1	Rm uncharacterized	7.54
XM_037414578.1	Rm uncharacterized	7.15
XR_005110507.1	Rm uncharacterized	7.03
XM_037423332.1	Rm myosin light chain 1-like	6.85
XM_037434134.1	Rm alpha-crystallin A chain-like	6.66
XM_037417989.1	Rm lysosomal acid glucosylceramindase-like	5.92
XM_037425789.1	Rm uncharacterized	5.25
XM_037413837.1	Rm antimicrobial peptide microplusin	5.18
XM_037421386.1	Rm cytosolic non-specific dipeptidase-like	5.10
XM_037427360.1	Rm venom protein 302-like	4.94
XM_037423526.1	Rm beta-secretase-like	4.58
XM_037421563.1	Rm choline/ethanolamine kinase-like	4.37
XM_037422021.1	Rm endochitinase-like	4.22
XM_037421388.1	Rm cytosolic non-specific dipeptidase-like	3.76
XM_037432624.1	Rm nucleoredoxin-like protein 2	3.73
XM_037412372.1	Rm activated RNA polymerase II transcriptional coactivator p15-like	3.68

**Table 4:** Upregulated hemocyte genes following the *B. bovis* infection, grouped by biological process, molecular function, and cellular component categories defined by high-level GO terms

	N	High-level GO category	Genes
Biological process	7	GO:0044238 primary metabolic process GO:0071704 organic substance metabolic process	XM_037412372.1 XM_037416570.1 XM_037417989.1 XM_037422021.1 XM_037422759.1 XM_037423526.1 XM_037435231.1
	6	GO:0044237 cellular metabolic process	XM_037412372.1 XM_037416570.1 XM_037417989.1 XM_037421563.1 XM_037422759.1 XM_037435231.1
	5	GO:0065007 biological regulation	XM_037412372.1 XM_037421514.1 XM_037427360.1 XM_037428883.1 XM_037432624.1
	5	GO:0006807 nitrogen compound metabolic process	XM_037412372.1 XM_037416570.1 XM_037417989.1 XM_037423526.1 XM_037435231.1
	4	GO:0050789 regulation of biological process	XM_037412372.1 XM_037421514.1 XM_037427360.1 XM_037428883.1
	3	GO:0009058 biosynthetic process	XM_037412372.1 XM_037416570.1 XM_037435231.1
	3	GO:0050794 regulation of cellular process	XM_037412372.1 XM_037427360.1 XM_037428883.1
	2	GO:0032501 multicellular organismal process GO:0003008 system process	XM_037421514.1 XM_037432624.1
	2	GO:0050896 response to stimulus	XM_037413837.1 XM_037428883.1
	2	GO:0071840 cellular component organization or biogenesis GO:0016043 cellular component organization	XM_037424720.1 XM_037427360.1
	1	GO:0023052 signaling GO:0051716 cellular response to stimulus	XM_037428883.1
	1	GO:0040007 growth GO:0016049 cell growth GO:0040008 regulation of growth	XM_037427360.1
	1	GO:0048518 positive regulation of biological process GO:0009893 positive regulation of metabolic process GO:0019222 regulation of metabolic process GO:0006367 transcription initiation from RNA polymerase II promoter GO:0045944 positive regulation of transcription by RNA polymerase II	XM_037412372.1
	1	GO:0051179 localization GO:0009056 catabolic process GO:0033036 macromolecule localization GO:0051234 establishment of localization GO:0032309 icosanoid secretion GO:0040008 regulation of growth GO:0050482 arachidonic acid secretion GO:0071715 icosanoid transport GO:1903963 arachidonate transport GO:0015908 fatty acid transport GO:0015909 long-chain fatty acid transport GO:0015718 monocarboxylic acid transport	XM_037422759.1
	1	GO:0006950 response to stress GO:0002376 immune system process GO:0006955 immune response GO:0045087 innate immune response GO:0042742 defense response to bacterium GO:0009605 response to external stimulus GO:0009607 response to biotic stimulus GO:0044419 biological process involved in interspecies interaction between organisms GO:0051707 response to another organism	XM_037413837.1
	1	GO:0051239 regulation of multicellular organismal process GO:0003012 muscle system process GO:0006936 muscle contraction	XM_037421514.1
	1	GO:0065008 regulation of biological quality GO:0048871 multicellular organismal homeostasis GO:0060249 anatomical structure homeostasis GO:0001894 tissue homeostasis	XM_037432624.1
Molecular function	6	GO:0005515 protein binding	XM_037413134.1 XM_037417133.1 XM_037424720.1 XM_037427360.1 XM_037427452.1 XM_037428883.1
	6	GO:0043167 ion binding	XM_037413837.1 XM_037420308.1 XM_037421386.1 XM_037423332.1 XM_037426593.1 XM_037434134.1
	4	GO:0016787 hydrolase activity	XM_037417989.1 XM_037421386.1 XM_037422759.1 XM_037423526.1
	3	GO:0098772 molecular function regulator activity	XM_037417133.1 XM_037427452.1 XM_037428883.1
	2	GO:0004866 endopeptidase inhibitor activity GO:0061135 endopeptidase regulator activity GO:0030414 peptidase inhibitor activity GO:0030234 enzyme regulator activity	XM_037417133.1 XM_037427452.1
	2	GO:0005198 structural molecule activity GO:0003735 structural constituent of ribosome	XM_037416570.1 XM_037435231.1
	1	GO:0016530 metallochaperone activity GO:0016531 copper chaperone activity	XM_037413837.1
	1	GO:0016740 transferase activity	XM_037421563.1
	1	GO:0030246 carbohydrate binding	XM_037422021.1
	1	GO:0030545 signaling receptor regulator activity GO:0048018 receptor ligand activity	XM_037428883.1
	1	GO:0097159 organic cyclic compound binding GO:1901363 heterocyclic compound binding	XM_037412372.1
	1	GO:0097367 carbohydrate derivative binding	XM_037422021.1
Cellular component	5	GO:0043226 organelle	XM_037412372.1 XM_037416570.1 XM_037421514.1 XM_037421516.1 XM_037435231.1
	4	GO:0005576 extracellular region	XM_037413837.1 XM_037417133.1 XM_037427360.1 XM_037427452.1
	4	GO:0016020 membrane	XM_037412258.1 XM_037422021.1 XM_037423526.1 XM_037428883.1
	4	GO:0043228 non-membrane-bounded organelle GO:0043232 intracellular non-membrane-bounded organelle	XM_037416570.1 XM_037421514.1 XM_037421516.1 XM_037435231.1
	2	GO:0030016 myofibril GO:0030017 sarcomere GO:0043292 contractile fiber GO:0032991 protein-containing complex GO:0015629 actin cytoskeleton GO:0099081 supramolecular polymer GO:0099512 supramolecular fiber	XM_037421514.1 XM_037421516.1
	2	GO:0005615 extracellular space	XM_037417133.1 XM_037427452.1
	1	GO:0043227 membrane-bounded organelle	XM_037412372.1

N: the number of upregulated hemocyte genes that fall under a similar GO term

**Fig. 7:**
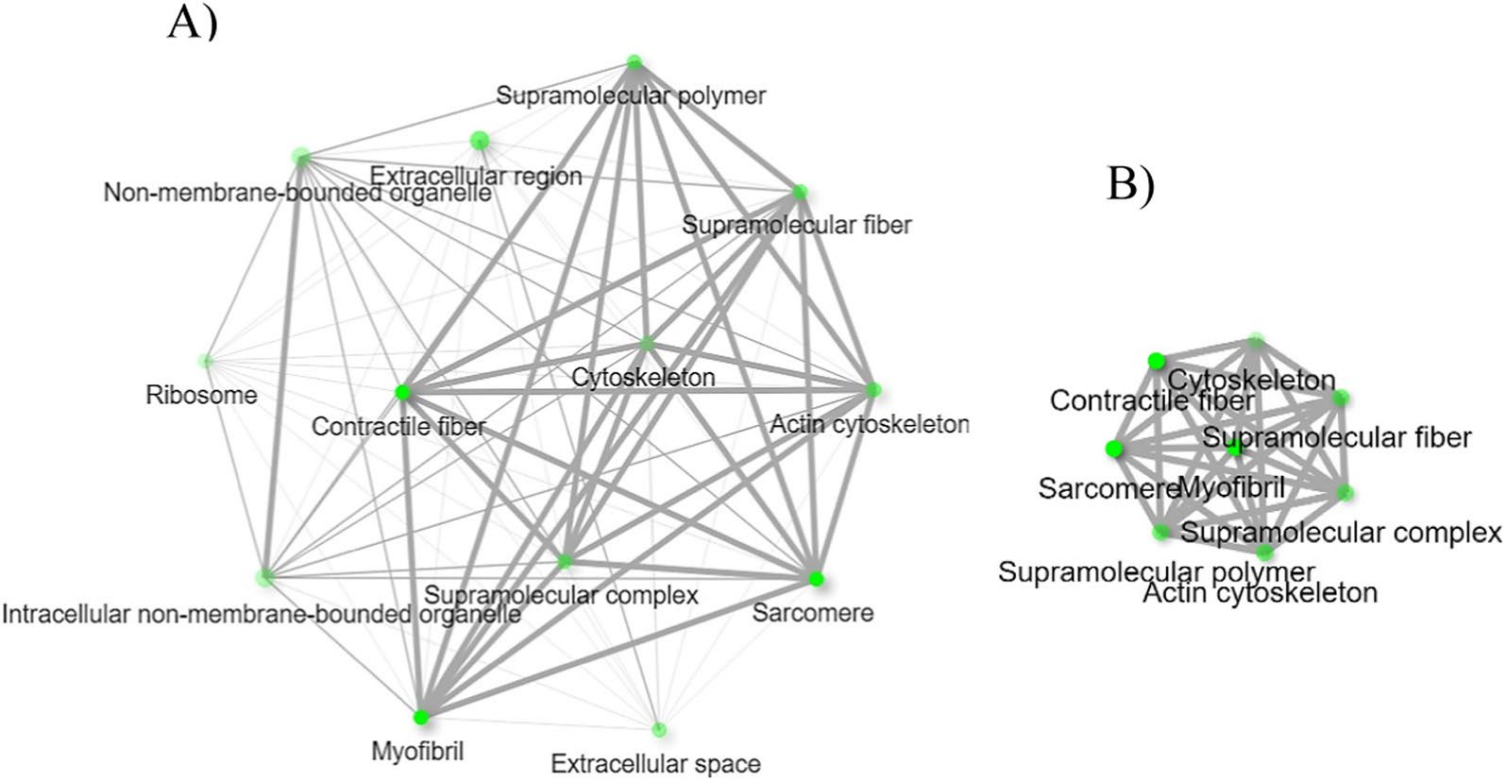
Cellular component pathways related to upregulated hemocyte genes following *B. bovis* infection. **A** Network of all the cellular component pathways and **B** cellular component pathways involved in cytoskeleton remodeling. Each node of the network represents an enriched GO term and the related GO terms are connected by a line. The thickness of the line reflects the percent of overlapping genes, and the size of the node corresponds to the number of genes

Remodeling of the cytoskeleton was previously observed in *I. scapularis* infected with *Anaplasma phagocytophilum* [36]. The findings of the study by Cabezas-Cruz et al. 2017 suggested cytoskeleton remodeling assisted pathogen infection and dissemination, and the infection affected tubulin, actin, septin, actin-related proteins, and motor proteins of ticks. Upregulation of the cytoskeletal protein spectrin α-chain, which stabilizes plasma membranes and organizes the organelles of cells, was observed in the midgut of *I. scapularis* upon infection with *A. phagocytophilum* owing to cytoskeleton rearrangement [37]. Another study suggested that *I. scapularis* remodeled its cytoskeleton in response to *A. phagocytophilum* infection by altering the ratio between monomeric globular G actin and filamentous F actin along with selective regulation of gene transcription in association with the RNA polymerase II [38]. This current RNA-Seq study shows highly regulated gene expression of cytoskeletal constituents of *R. microplus* hemocytes in response to *B. bovis* infection along with the upregulation of activated RNA polymerase II transcriptional coactivator p15-like (XM_037412372.1). RNA polymerase II transcriptional coactivator p15-like acts in organelles (Table 4) and positively regulates biological process pathways (Table 4). This study indicates the possible cytoskeletal rearrangement in *R. microplus* hemocytes that may have taken place in response to *Babesia* infection. The network of cellular component pathways involving cytoskeleton remodeling are shown in Fig. 7A and B, respectively.

While the pathogen regulates tick factors such as cytoskeletal arrangement, ticks recruit innate immune molecules to defend against the pathogen. *Rhipicephalus microplus* hemocytes upregulated the expression of leucine-rich repeat-containing protein 20-like (XM_037413134.1) by 129.0-fold (Table 3) in response to *B. bovis* infection. This gene was recognized in pathways involving protein binding (Table 4). Leucine-rich repeat-containing proteins serve as innate immune proteins that sense pathogen-associated molecular patterns and have been previously recognized in the innate immune response of mosquitoes, which mediate antiplasmodium immunity [39, 40]. The leucine-rich repeat-containing protein of *Haemaphysalis longicornis* was found to have receptor–ligand interaction with *B. microti* [41]. The same study also indicated the downregulation of the gene expression of AMPs upon the knockdown of *H. longicornis* leucine-rich repeat-containing protein [41], suggesting the immune modulation mediated by this gene. This current RNA-Seq study reports the upregulation of *R. microplus* antimicrobial peptide microplusin (XM_037413837.1), unique to *B. bovis* infection (Table 4). These findings suggest that *B. bovis* may be recognized by *R. microplus* hemocytes by receptor-ligand interaction through leucine-rich repeat-containing protein 20-like during its innate immune response.

**Fig. 8:**
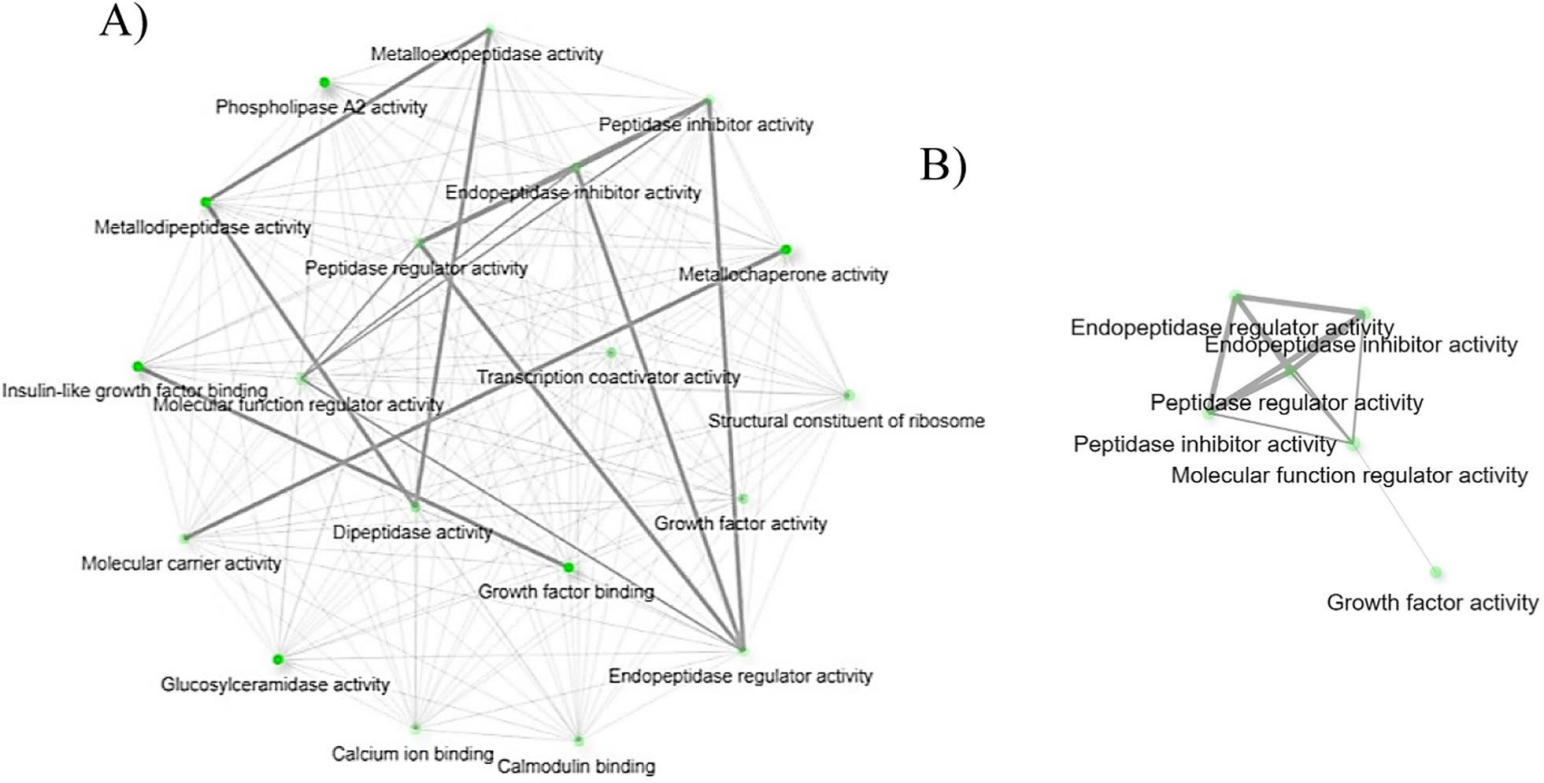
Molecular functional pathways related to upregulated hemocyte genes following *B. bovis* infection. **A** Network of all the molecular functional pathways and **B** molecular functional pathways involved in peptidase inhibition activity. Each node of the network represents an enriched GO term and the related GO terms are connected by a line. The thickness of the line reflects the percent of overlapping genes, and the size of the node corresponds to the number of genes

Tick-borne pathogens often display metalloproteases [29] that may be involved in the digestion of hemolymph proteins and bind to danger-sensing receptors for their destruction [42]. However, the expression of endopeptidase inhibitors in tick hemolymph protects ticks against the proteases of invading pathogens and plays a significant role in phagocytosis [28]. The current RNA-Seq study found two genes associated with endopeptidase inhibitor activities (Table 4) unique to *B. bovis* infection, namely, complement C3-like (XM_037417133.1) and *Austrelaps superbus* venom factor 1-like (XM_037427452.1), which were upregulated 8.0-fold and 8.0-fold, respectively (Table 3). Complement C3 protein activates the complement system upon microbial invasion cleaving into C3b and C3a in the complement system response [43]. A previous study indicated that phagocytosis in *I. ricinus* infected with *Metarhizium robertsii* was assisted by C3-like complement components and α2-macroglobulin pan-protease inhibitors secreted into the hemolymph [44]. *A. superbus* venom factor 1 is similar in structure and function to the complement component C3b [45]. However, the functional role of *A. superbus* venom factor 1 in the tick innate immune response is unknown. The network of molecular function pathways (Fig. 8A and B) identified among upregulated hemocyte genes during *B. bovis* infection suggests that there is a defense mechanism of *R. microplus* hemocytes against *B. bovis* parasites consisting of peptidase inhibitors.

**Fig. 9:**
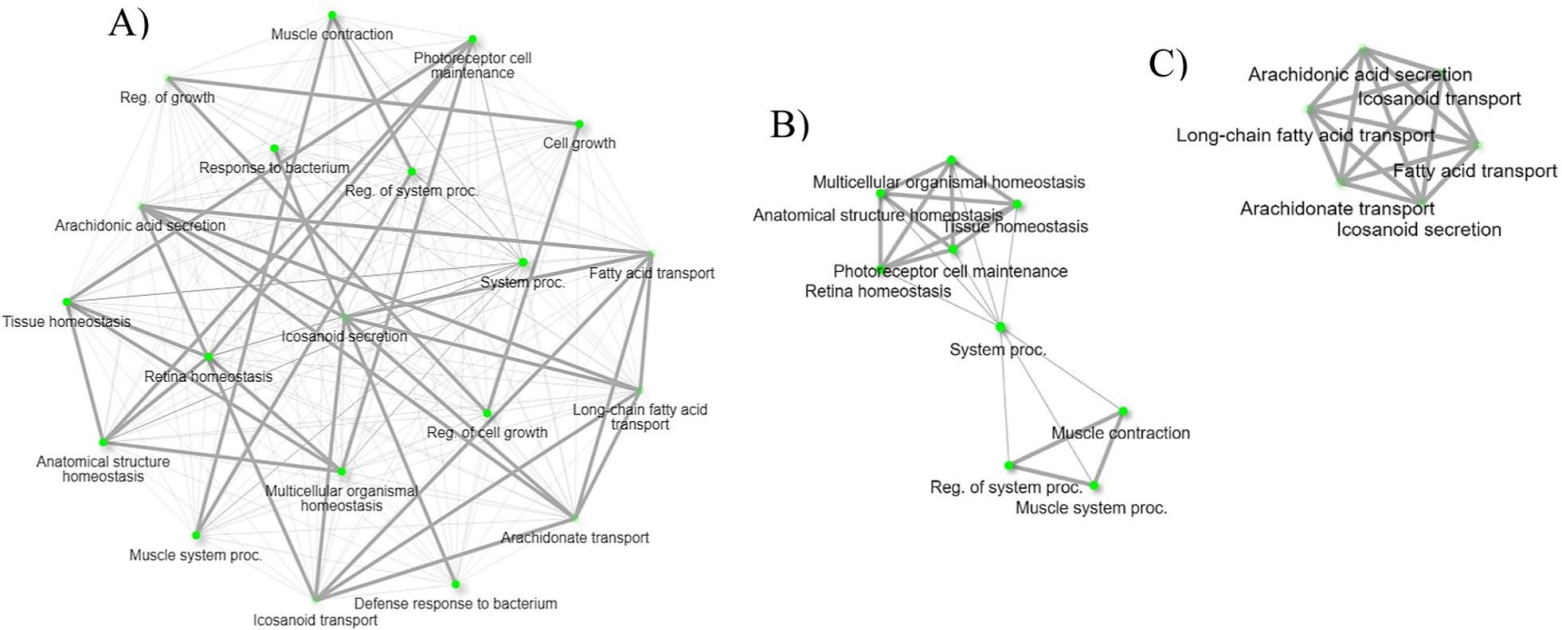
Biological process pathways related to upregulated hemocyte genes following *B. bovis* infection. **A** Network of all the biological process pathways, **B** biological process pathways involved in homeostasis, and **C** arachidonic acid and eicosanoid secretion and transportation. Each node of the network represents an enriched GO term and the related GO terms are connected by a line. The thickness of the line reflects the percent of overlapping genes, and the size of the node corresponds to the number of genes

The upregulated genes of biological processes during *B. bovis* infection also include several genes that control arthropod immunity. Arachidonic acid and eicosanoid secretion and transportation pathways were found among upregulated genes unique to *B. bovis* infection (Table 4). Arachidonic acid is a primary precursor for the production of eicosanoids, which are involved in cellular immune responses of insects, including phagocytosis, nodulation, hemocyte spreading, and microaggregation [46]. Previously it was shown that the protozoan parasite, *Trypanosoma rangeli*, suppresses phagocytosis in *Rhodnius prolixus* by affecting eicosanoid biosynthesis [47]. An uncharacterized *R. microplus* gene, XM_037422759.1 (9.0-fold increase), was identified in the biological process pathways of arachidonic acid and eicosanoid secretion and transportation (Table 4). The network of biological process pathways of arachidonic acid and eicosanoid secretion and transportation found in this study are illustrated in Fig. 9A and C. Further, previous insect studies have shown the interplay between arachidonic acid and homeostasis, where arachidonic acid acts as the precursor for signaling molecules [48]. This RNA-Seq study identified nucleoredoxin-like protein 2 (XM_037432624.1) and venom protein 302-like (XM_037427360.1) among upregulated genes in response to *B. bovis* infection (Table 3) and found them in the biological pathways of tissue homeostasis and regulation of growth, respectively (Table 4). The network of biological processes of tissue homeostasis and regulation of growth is illustrated in Fig. 9A and B. This finding suggests that the tick defense unique to *B. bovis* infection may be equipped with eicosanoids, which warrant further investigations.

### Hemocyte gene upregulation restricted to *B. bigemina* infection

The upregulation of 26 tick genes was limited to *B. bigemina* infection (Table 5). Out of the 26 upregulated genes, 22 were identified as protein-coding genes, 3 were lncRNAs, and 1 was not found by the Ensemble annotation system [22]. Differentially regulated hemocyte genes upon *B. bigemina* infection showed the upregulation fold ranged between 3.0-fold and 655.0-fold for protein-coding genes and between 145.0-fold and 27.0-fold for putative lncRNAs (XR_005109643.1, XR_005108421.1, and XR_005108101.1) (Table 5). *B. bigemina* infection primarily resulted in a high transcriptional level of genes associated with cellular metabolic process pathways (Table 6). Salivary cystatin-L2-like (XM_037434630.1) was upregulated 225.0-fold and found to be associated with negative regulation of biological processes (Table 6). Salivary cystatins are cathepsin inhibitors and recognized as one of the major host immune system modulators [49]. However, within the ticks, cystatins contribute to pathogen survival and transmission [50] and innate immune response through the inhibition of cysteine protease [49]. It was evident that *H. longicornis* cystatin-2 played a role in its innate immunity through its ability to inhibit the growth of *B. bovis *in vitro [51]. Moreover, the high transcription level of *H. longicornis* cystatin-2 was found in *Babesia gibsoni* infected tick larvae [51], suggesting the involvement of cystatin-2 in ticks controlling *Babesia* infection. The highly elevated level of *R. microplus* salivary cystatin-L2-like found in this study may be linked to controlling *B. bigemina* growth in hemolymph. Control machinery against *B. bigemina* parasites in *R. microplus* hemolymph may be loaded with antimicrobial peptides since *B. bigemina* infection resulted in high expression of acanthoscurrins. Acanthoscurrin-2-like transcript variant X1 (XM_037413525.1) and acanthoscurrin-2-like (XM_037416561.1) were upregulated 230.0-fold and 48.0-fold, respectively. Acanthoscurrins are shown to be synthesized in the hemocytes of arachnids and released upon immune challenge [52]. The substantial upregulation of acanthoscurrins found in the current study may be specific to *B. bigemina* infection, as indicated by recent findings regarding the pathogen-specific roles of AMPs in *Drosophila* [53].

**Table 5:** Tick genes upregulated in response to *B. bigemina* infection only with an FDR < 0.05

Accession no.	Gene	Fold increase
XM_037429235.1	Rm uncharacterized	655.63
XM_037429314.1	Rm uncharacterized	529.74
XM_037427095.1	Rm uncharacterized	463.87
XM_037413525.1	Rm acanthoscurrin-2-like transcript variant X1	230.43
XM_037425236.1	Rm mite group 2 allergen-like Ixo r 2	225.66
XM_037434630.1	Rm salivary cystatin-L2-like	225.03
XM_037429546.1	Rm uncharacterized	167.56
XM_037422229.1	Rm uncharacterized	163.37
XR_005108101.1	Rm uncharacterized	145.48
XR_005109643.1	Rm uncharacterized	132.42
XM_037422880.1	Rm NPC intracellular cholesterol transported 2-like	68.77
XM_037428968.1	Rm uncharacterized	61.94
XM_037432251.1	Rm heat shock protein 68-like	61.75
XM_037416561.1	Rm acanthoscurrin-2-like	48.04
XM_037417508.1	Rm glycine-rich protein DOT1-like	35.14
XM_037414515.1	Rm glycine-rich cell wall structural protein 2-like	28.03
XR_005108421.1	Rm uncharacterized	26.85
XM_037413852.1	Rm uncharacterized	22.08
XM_037416559.1	Rm glycine-rich protein DOT1-like	17.31
XM_037423271.1	Rm pyruvated kinase PKM-like	11.79
XM_037421303.1	Rm acid ceramidase-like	11.40
XM_037413850.1	Rm uncharacterized	6.75
XM_037422694.1	Rm normal mucose of esophagus-soecific gene 1 protein-like	5.90
XM_037418947.1	Rm protein Skeletor transcript variant X2	3.60
XM_037429406.1	Rm extensin-like	3.32
XM_037428695.1	Rm peptide methionine sulfoxide reductase-like	3.03

**Table 6:** Upregulated hemocyte genes following the *B. bigemina* infection, grouped by biological process and cellular component categories defined by high-level GO terms

	N	High-level GO category	Genes
Biological process	3	GO:0044237 cellular metabolic process GO:0044238 primary metabolic process GO:0071704 organic substance metabolic process GO:0032787 monocarboxylic acid metabolic process	XM_037421303.1 XM_037423271.1 XM_037434630.1
	2	GO:0006807 nitrogen compound metabolic process	XM_037423271.1 XM_037434630.1
	1	GO:0048519 negative regulation of biological process GO:0050789 regulation of biological process GO:0019222 regulation of metabolic process GO:0050794 regulation of cellular process GO:0065009 regulation of molecular function	XM_037434630.1
	1	GO:0006457 protein folding	XM_037432251.1
	1	GO:0016052 carbohydrate catabolic process GO:0006096 glycolytic process GO:0006757 ATP generation from ADP GO:0046031 ADP metabolic process GO:0046939 nucleotide phosphorylation GO:0006090 pyruvate metabolic process	XM_037423271.1
Cellular component	1	GO:0000323 lytic vacuole GO:0005764 lysosome GO:0005773 vacuole	XM_037421303.1

N: the number of upregulated hemocyte genes that fall under a similar GO term

Hemocyte defense pathways against *B. bigemina* parasites are not known, but they may consist of known gene candidates to date. Mite group 2 allergen-like Ixo r 2 (XM_037425236.1) from *R. microplus* hemocytes is such a candidate, as it was triggered and highly differentially expressed (226.0-fold) upon the infection. The group 2 allergen-like protein is found to be expressed in the tick midgut and hemolymph following the blood meal and constitutively expressed in all the life stages of a tick [54]. The interaction of the putative lipid-binding domain of group 2 allergen-like protein and IgE shows its effect in immune signaling pathways [54]. It is valuable to pursue an understanding of the tick defense pathways and the role of group 2 allergen-like proteins against *B. bigemina* parasites for the further advancement of information at the tick–*Babesia* interface.

Another class of protective proteins of ticks that are expressed following the blood meal are heat shock proteins (HSPs) [55]. These stress response proteins are primarily involved in protein homeostasis, which prevents the accumulation and aggregation of misfolded proteins [56]. *Babesia bigemina* infection upregulated heat shock protein 68-like (XM_037432251.1) by 62.0-fold and was found in the biological process pathways of protein folding (Table 6). Invading pathogens endure stress in the host including nutritional starvation and oxidative stress and their survival and proliferation depend on protein homeostasis [57]. Similarly, the infected, stressed host cell acts to maintain its protein homeostasis while activating the immune response pathways [57]. *Ixodes scapularis* midguts and salivary glands infected with *A. phagocytophilum* show the upregulation of HSP20 and the downregulation of HSP70 [55], suggesting their influence on the bacterial growth and survival. However, the proteomic regulation underlying the effects caused by *R. microplus* HSP68-like reported in this study is unknown, and it may favor the hemocytes or *B. bigemina*, or both, to defeat each other.

**Fig. 10:**
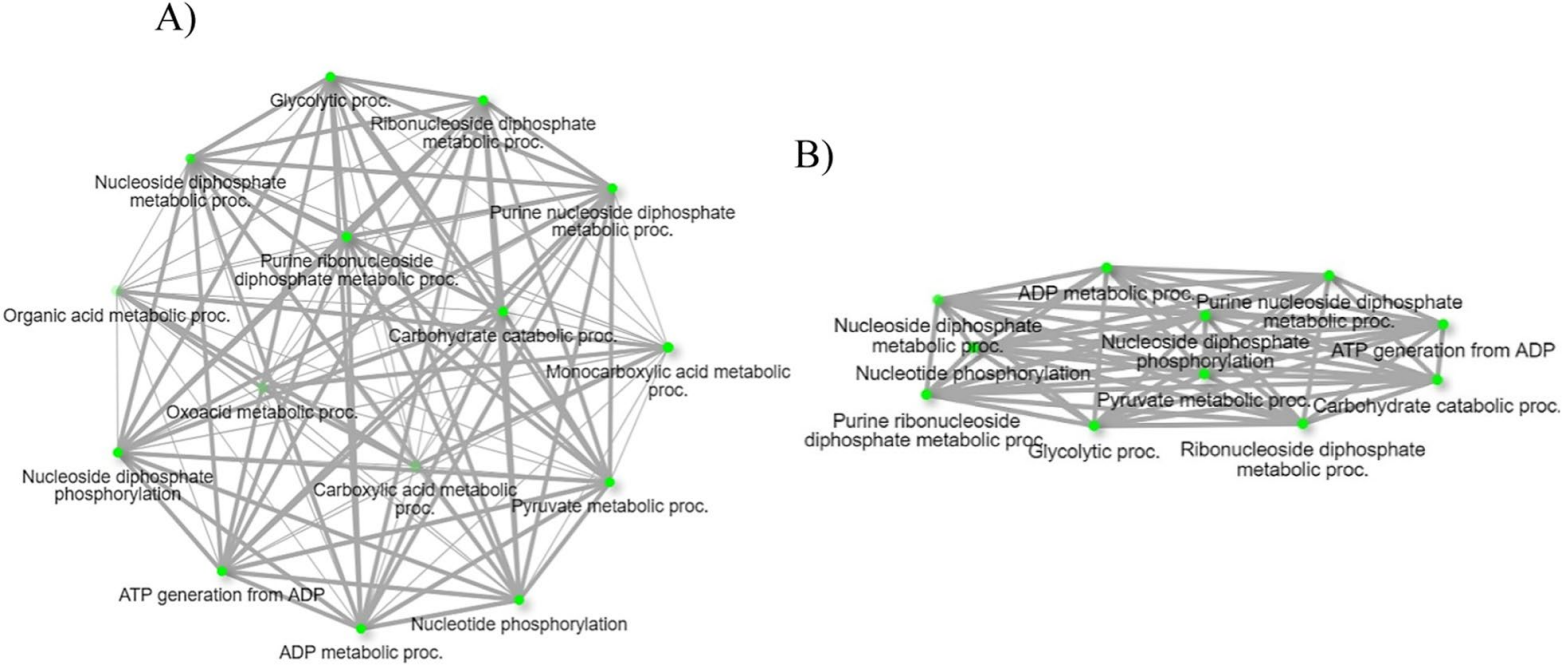
Biological process pathways related to upregulated hemocyte genes following *B. bigemina* infection. **A** Network of all the biological process pathways, and **B** biological process pathways involved in glycolytic process. Each node of the network represents an enriched GO term and the related GO terms are connected by a line. The thickness of the line reflects the percent of overlapping genes, and the size of the node corresponds to the number of genes

The activation of the immune response in hemolymph consumes energy in the form of glucose. Therefore, glycolysis plays a key role in the immune response of insects [58]. Pyruvate kinase (PKM) is a critical enzyme that acts in the final step of glycolysis to produce ATP [59]. This RNA-Seq study reports on the differential regulation of pyruvate kinase PKM-like (XM_037423271.1) in the hemocytes infected with *B. bigemina*, suggesting the modulation of glycolytic processes (Table 6) in tick physiology on the basis of ongoing infection. The network of glycolytic processes found among upregulated genes upon *B. bigemina* infection is shown in Fig. 10A and B. Like glycolytic processes, *B. bigemina* infection also regulated the synthesis of sphingosine and fatty acid in tick hemocytes through the differential expression of acid ceramidase-like (XM_037421303.1). Acid ceramidase breaks down ceramide into sphingosine and fatty acid and is found in the lysosome or lytic vacuole (Table 6). Sphingolipids that include ceramide and sphingosine are known as essential for the cellular homeostasis of insects [60], and they mediate apoptosis [61]. Differential regulation of *R. microplus* acid ceramidase indicates the possible maintenance of cellular homeostasis in response to *B. bigemina* infection.

### Hemocyte gene downregulation occurs mutually in *B. bovis *and* B. bigemina* infections

**Fig. 11:**
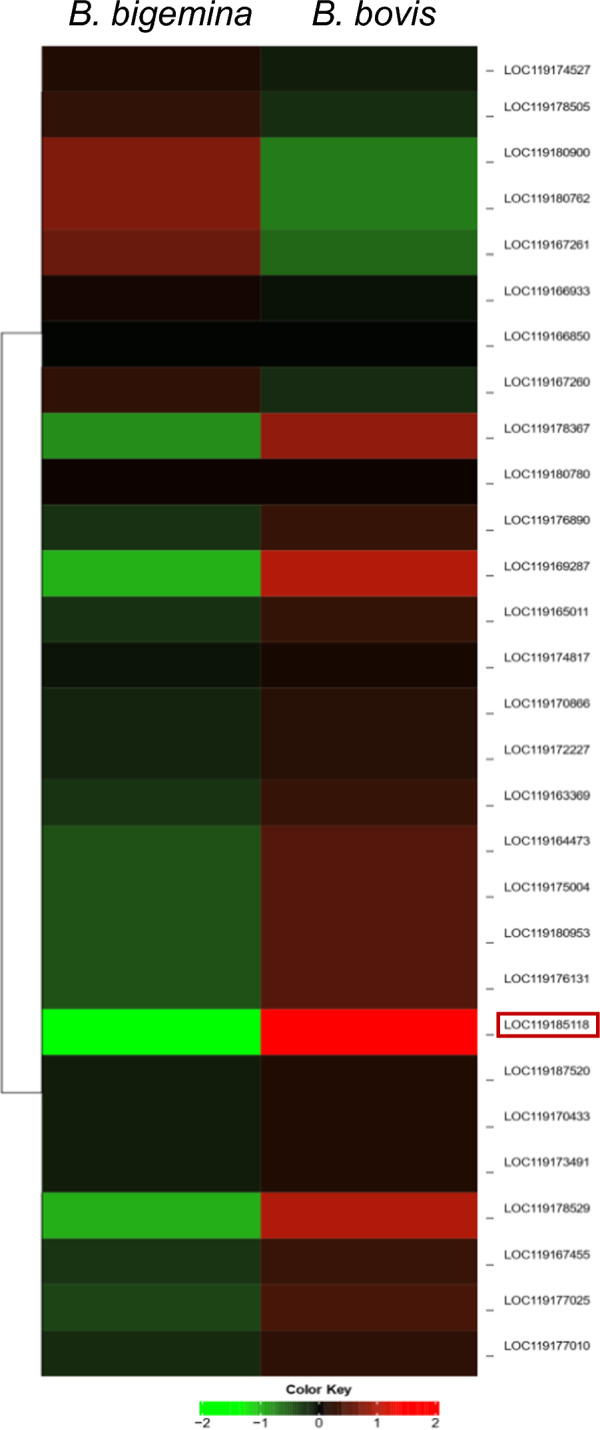
Hierarchical clustering of downregulated hemocyte gene expression following *Babesia* infection. The red frame refers to matrix metalloproteinase-14-like variant X1 (XM_037434231.1) that was identified in the cellular component pathways of the external encapsulating structure of the extracellular matrix

We found that 30 tick genes were downregulated regardless of the infecting *Babesia* species. Out of the 30 downregulated genes, 28 were identified as protein-coding genes, 1 was lncRNA, and 1 was not found by the Ensembl annotation system [22]. The hierarchical clustering of log fold-change of downregulated hemocyte gene expression suggests that the differential regulation of genes in response to *B. bovis* infection varies from that of *B. bigemina* infection (Fig. 11). Protein-coding genes were downregulated at a range between 4.0-fold and 232.0-fold. The putative lncRNA (XR_005111866.1) was downregulated by 151.0-fold and 119.0-fold in response to *B. bovis* and *B. bigemina,* respectively. The fold decrease in hemocyte genes was higher in response to *B. bovis* than *B. bigemina* infection (Table 7). Matrix metalloproteinase-14-like variant X1 (XM_037434231.1) showed a substantial difference (highlighted in the heatmap) in expression (*B. bovis* 22.0-fold and *B. bigemina* 7.0-fold) and was identified in the cellular component pathways of the external encapsulating structure of the extracellular matrix (Table 8).

**Table 7:** Tick genes downregulated in response to *Babesia* infections with an FDR < 0.05

Accession no.	Gene symbol	Gene	Fold decrease	
			*B. bovis*	*B. bigemina*
XM_037422147.1	LOC119170866	Rm homeodomain-interacting protein kinase 2-like	232.56	183.2
XR_005111866.1	LOC119187520	Rm uncharacterized	151.27	119.16
XM_037426165.1	LOC119175004	Rm terminal nucleotidyltransferase 5C-like	74.65	39.32
XM_037420399.1	LOC119169287	Rm flavin-containing monooxygenase 5-like	56.31	14.1
XM_037429734.1	LOC119178529	Rm hemocytin-like	35.66	9.14
XM_037429567.1	LOC119178367	Rm E-selectin-like	34.14	11.01
XM_037434231.1	LOC119185118	Rm matrix metalloproteinase-14-like variant X1	22.03	6.65
XM_037425473.1	LOC119174527	Rm endothelial transcription factor GATA-2-like	20.18	24.37
XM_037418224.1	LOC119166850	Rm spectrin beta chain-like	18.63	19.07
XM_037427293.1	LOC119176131	Rm ataxin-2-like protein	17.28	8.87
XM_037418323.1	LOC119166933	Rm bone morphogenetic protein 2-like	14.89	16.4
XM_037425904.1	LOC119174817	Rm importin subunit alpha-7-like	13.39	11.9
XM_037429710.1	LOC119178505	Rm juvenile hormone acid *O*-methyltransferase-like	12.36	17.58
XM_037434236.1	LOC119185118	Rm matrix metalloproteinase-14-like variant X2	12.32	5.98
XM_037418932.1	LOC119167455	Rm tyrosine-protein kinase receptor Tie-1-like	12.23	8.09
XM_037428358.1	LOC119177010	Rm clotting factor C-like	11.77	8.51
XM_037416668.1	LOC119164473	Rm bestrophin-4-like	11.41	6.55
XM_037417206.1	LOC119165011	Rm FYVE,RhoGEF and PH domain-containing protein 2-like	9.05	6.19
XM_037428376.1	LOC119177025	Rm uncharacterized	8.91	5.15
XM_037415351.1	LOC119163369	Rm dystonin-like	8.69	5.83
XM_037418712.1	LOC119167260	Rm leucine-rich repeat-containing protein 15-like	8.58	11.96
XM_037424296.1	LOC119173491	Rm solute carrier family 13 member 2-like	8.41	7
XM_037428235.1	LOC119176890	Rm Na(+)/H(+) exchanger beta-like	7.59	5.15
XM_037423261.1	LOC119172227	Rm gamma-butyrobetaine dioxygenase-like	7.22	5.55
XM_037418714.1	LOC119167261	Rm protein toll-like	7.17	16.36
XM_037431915.1	LOC119180780	Rm MAP kinase-interacting serine/threonine-protein kinase 1-like	7.04	6.68
XM_037432108.1	LOC119180953	Rm LIM domain and actin-binding protein 1-like	6.51	3.41
XM_037421596.1	LOC119170433	Rm nocturnin-like	6.29	5.18
XM_037432037.1	LOC119180900	Rm vitellogenin-2-like	4.95	13.38
XM_037431896.1	LOC119180762	Rm vitellogenin-5-like	4.27	11.44

**Table 8:** Downregulated hemocyte genes following the *Babesia* infection, grouped by biological process, molecular function, and cellular component categories defined by high-level GO terms

	N	High-level GO category	Genes
Biological process	7	GO:0006807 nitrogen compound metabolic process GO:0071704 organic substance metabolic process	XM_037421596.1 XM_037422147.1 XM_037423261.1 XM_037425473.1 XM_037428358.1 XM_037431915.1 XM_037434231.1
	6	GO:0051179 localization GO:0051234 establishment of localization GO:0006810 transport	XM_037416668.1 XM_037424296.1 XM_037425904.1 XM_037428235.1 XM_037431896.1 XM_037432037.1
	6	GO:0044238 primary metabolic process	XM_037421596.1 XM_037422147.1 XM_037425473.1 XM_037428358.1 XM_037431915.1 XM_037434231.1
	5	GO:0044237 cellular metabolic process	XM_037421596.1 XM_037422147.1 XM_037423261.1 XM_037425473.1 XM_037431915.1
	4	GO:0065007 biological regulation	XM_037418714.1 XM_037421596.1 XM_037425473.1 XM_037428235.1
	3	GO:0050789 regulation of biological process	XM_037418714.1 XM_037421596.1 XM_037425473.1
	3	GO:0033036 macromolecule localization	XM_037425904.1 XM_037431896.1 XM_037432037.1
	2	GO:0002376 immune system process GO:0050794 regulation of cellular process	XM_037418714.1 XM_037425473.1
	2	GO:0032501 multicellular organismal process GO:0019222 regulation of metabolic process	XM_037421596.1 XM_037425473.1
	2	GO:0009058 biosynthetic process	XM_037423261.1 XM_037425473.1
	2	GO:0010876 lipid localization GO:0006869 lipid transport	XM_037431896.1 XM_037432037.1
	1	GO:0023052 signaling GO:0050896 response to stimulus GO:0051716 cellular response to stimulus GO:0048583 regulation of response to stimulus GO:0002682 regulation of immune system process GO:0006955 immune response GO:0002221 pattern recognition receptor signaling pathway GO:0002224 toll-like receptor signaling pathway	XM_037418714.1
	1	GO:0032502 developmental process GO:0048513 animal organ development GO:0048856 anatomical structure development GO:0048534 hematopoietic or lymphoid organ development GO:0002520 immune system development GO:0030097 hemopoiesis	XM_037425473.1
	1	GO:0048511 rhythmic process	XM_037421596.1
	1	GO:0071840 cellular component organization or biogenesis GO:0016043 cellular component organization	XM_037415351.1
	1	GO:0032259 methylation	XM_037429710.1
	1	GO:0051641 cellular localization	XM_037425904.1
	1	GO:0065008 regulation of biological quality	XM_037428235.1
	1	GO:0006915 apoptotic process GO:0008219 cell death GO:0012501 programmed cell death	XM_037428376.1
Molecular function	9	GO:0043167 ion binding	XM_037415351.1 XM_037417206.1 XM_037420399.1 XM_037422147.1 XM_037423261.1 XM_037425473.1 XM_037431915.1 XM_037432108.1 XM_037434231.1
	8	GO:0005515 protein binding	XM_037415351.1 XM_037418224.1 XM_037418323.1 XM_037418712.1 XM_037418932.1 XM_037425904.1 XM_037429734.1 XM_037432108.1
	6	GO:0046872 metal ion binding	XM_037415351.1 XM_037417206.1 XM_037423261.1 XM_037425473.1 XM_037432108.1 XM_037434231.1
	5	GO:0005215 transporter activity	XM_037416668.1 XM_037424296.1 XM_037428235.1 XM_037431896.1 XM_037432037.1
	5	GO:0097159 organic cyclic compound binding GO:1901363 heterocyclic compound binding	XM_037420399.1 XM_037422147.1 XM_037425473.1 XM_037427293.1 XM_037431915.1
	5		
	4	GO:0016740 transferase activity	XM_037422147.1 XM_037426165.1 XM_037429710.1 XM_037431915.1
	4	GO:0016787 hydrolase activity	XM_037418714.1 XM_037421596.1 XM_037428358.1 XM_037434231.1
	3	GO:0022857 transmembrane transporter activity	XM_037416668.1 XM_037424296.1 XM_037428235.1
	3	GO:0036094 small molecule binding	XM_037420399.1 XM_037422147.1 XM_037431915.1
	3	GO:0008092 cytoskeletal protein binding	XM_037415351.1 XM_037418224.1 XM_037432108.1
	2	GO:0003779 actin binding	XM_037418224.1 XM_037432108.1
	2	GO:0098772 molecular function regulator activity	XM_037417206.1 XM_037418323.1
	2	GO:0005319 lipid transporter activity	XM_037431896.1 XM_037432037.1
	2	GO:0097367 carbohydrate derivative binding	XM_037422147.1 XM_037431915.1
	1	GO:0005198 structural molecule activity GO:0005200 structural constituent of cytoskeleton GO:0008289 lipid binding	XM_037418224.1
	1	GO:0060089 molecular transducer activity GO:0038023 signaling receptor activity	XM_037418714.1
	1	GO:0003700 DNA-binding transcription factor activity	XM_037425473.1
	1	GO:0005085 guanyl-nucleotide exchange factor activity GO:0030234 enzyme regulator activity	XM_037417206.1
	1	GO:0030545 signaling receptor regulator activity GO:0030546 signaling receptor activator activity GO:0048018 receptor ligand activity	XM_037418323.1
	1	GO:0044877 protein-containing complex binding	XM_037432108.1
Cellular component	3	GO:0016020 membrane	XM_037418714.1 XM_037424296.1 XM_037428235.1
	3	GO:0043226 organelle	XM_037418224.1 XM_037425473.1 XM_037428376.1
	2	GO:0043227 membrane-bounded organelle	XM_037425473.1 XM_037428376.1
	1	GO:0032991 protein-containing complex GO:0005938 cell cortex GO:0043228 non-membrane-bounded organelle	XM_037418224.1
	1	GO:0031012 extracellular matrix GO:0030312 external encapsulating structure	XM_037434231.1

N: the number of upregulated hemocyte genes that fall under a similar GO term

Matrix metalloproteinases (MMPs) are linked to the tissue remodeling process upon infections with protozoan parasites, including *Plasmodium falciparum*, *Trypanosoma brucei*, *Toxoplasma gondii*, and *Leishmania tropica* [62]. MMPs initiate the tissue repairing or remodeling process right after the infection is cleared [63]. During malaria infection of red blood cells, it was suggested that the parasite-derived product modulates the expression of MMPs and endogenous MMP inhibitors [62]. MMPs are endopeptidases [64], and the MMPs found in this study may be accompanied by elevated levels of endopeptidase inhibitors found among upregulated hemocyte genes (Tables 2 and 4) in response to *Babesia* infection. The presence of MMPs observed in this study indicated the formation of an external encapsulating structure on the extracellular matrix (Table 8) upon the *Babesia* infection. Encapsulation is a defense mechanism that host arthropods use to respond to parasites. Hemocytes surround and adhere to the surface of the parasite forming a capsule-like envelope, together with melanin, to kill the parasite [65]. This RNA-Seq finding suggests that the *R. microplus* hemocyte has the potential to encapsulate *B. bovis* and *B. bigemina*.

The biological process pathways primarily detected among downregulated hemocyte genes in response to *Babesia* infection are from the tick immune response. Endothelial transcription factor GATA-2-like (XM_037425473.1), identified in the membrane-bounded organelle (Table 8), was found in the pathways, which include immune system development through hematopoietic and lymphoid organ development and hemopoiesis (Table 8). GATA transcription factors are critical for hematopoiesis in insects and in *Drosophila*, and GATA mediates cell differentiation to produce plasmatocytes and crystal cells [66]. The downregulation upon *Babesia* infection (*B. bovis* 20.0-fold and *B. bigemina* 24.0-fold) suggests that hematopoiesis may be interrupted during the parasitic infection. Uncharacterized gene XM_037428376.1 from *R. microplus*, identified in the apoptotic process or programmed cell death pathways, is also differentially regulated, similarly to *R. microplus* GATA-2 (Table 7). Apoptosis, or programmed cell death, is a vital process for the development and functioning of the immune system, as it regulates hematopoietic homeostasis [67].

**Fig. 12:**
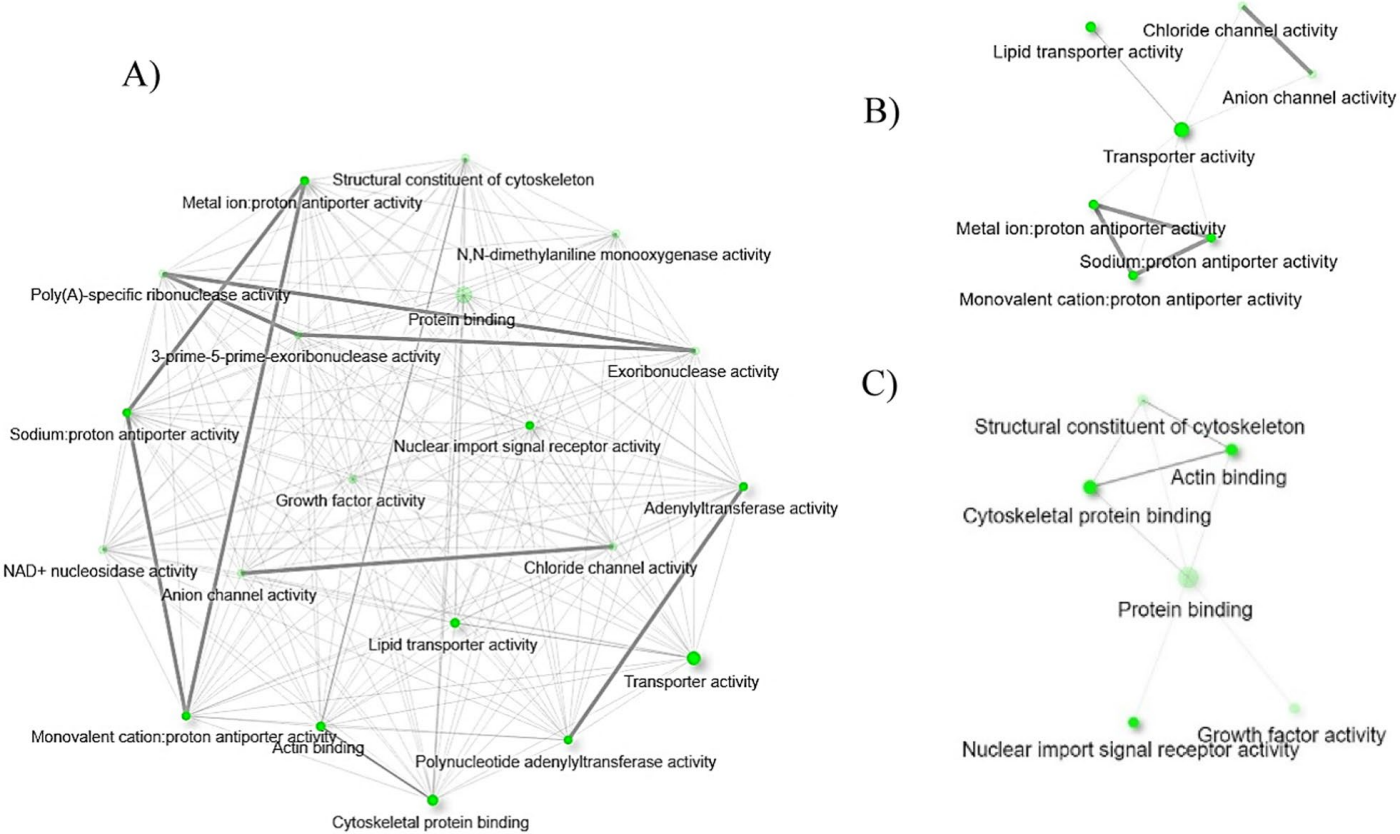
Molecular functional pathways related to downregulated hemocyte genes following *Babesia* infection. **A** Network of all the molecular functional pathways, **B** molecular functional pathways involved in transporter activity, and **C** cytoskeleton proteins. Each node of the network represents an enriched GO term and the related GO terms are connected by a line. The thickness of the line reflects the percent of overlapping genes, and the size of the node corresponds to the number of genes

**Fig. 13:**
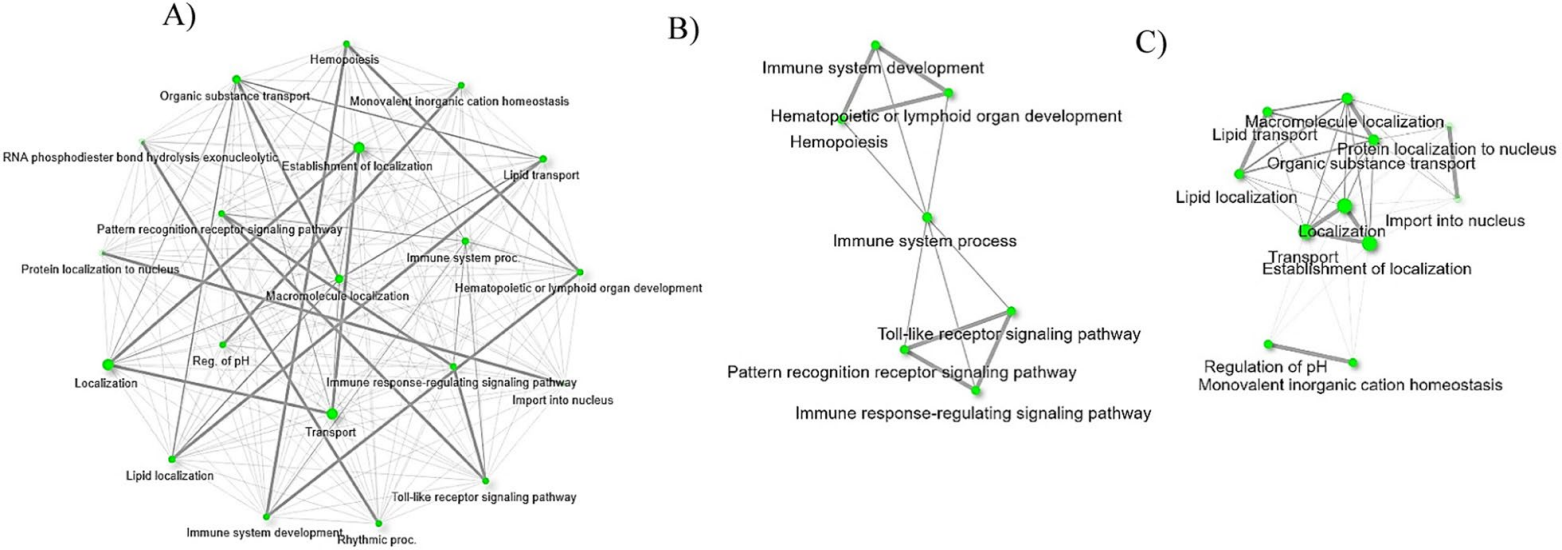
Biological process pathways related to downregulated hemocyte genes following *Babesia* infection. **A** Network of all the biological process pathways, **B** biological process pathways involved in the tick immune system, and **C** lipid transport and localization. Each node of the network represents an enriched GO term and the related GO terms are connected by a line. The thickness of the line reflects the percent of overlapping genes, and the size of the node corresponds to the number of genes

This RNA-Seq study revealed the downregulation of multiple immune genes during the infection with *Babesia*. *Rhipicephalus microplus* hemocytin (XM_037429734.1) was differentially regulated during *B. bovis* infection (36.0-fold decrease) and *B. bigemina* infection (9.1-fold decrease). Hemocytin is an immune recognition glycoprotein and an aggregation factor that is expressed and processed in hemocytes and plays a key role in coagulation, nodulation, and encapsulation [68, 69]. *Rhipicephalus microplus* hemocytin was found in the pathways involving protein binding (Table 8) and the network of molecular function pathways of protein binding was downregulated during *Babesia* infection, as shown in Fig. 12A and C. The tick immune gene, protein toll-like (XM_037418714.1), was identified in the membrane (Table 8), and in the pattern recognition receptor signaling pathway or toll-like receptor signaling pathway (Table 8) and downregulated upon *Babesia* infection (*B. bovis* 7.0-fold and *B. bigemina* 16.0-fold). This finding is consistent with the previous study that indicated the downregulation of most of the toll pathway components in *R. microplus* during infection with *A. marginale* [70]. The data suggest that *R. microplus* facilitates bacterial or protozoal colonization by dropping its immune response, favoring vector competency. The network of immune system pathways found among the downregulated genes in response to *Babesia* infection is shown in Fig. 13A and B.

The regulation of molecular metabolism during the insect immune response has been extensively studied [71]. The current study found the downregulation of multiple genes related to metabolism in *R. microplus* hemocytes in response to *Babesia* infection. Homeodomain-interacting protein kinase (Hipk) is a serine-threonine kinase that plays an important role in a multitude of cellular processes, including metabolism, regulation of cell proliferation, and apoptosis [72]. *Rhipicephalus microplus* Hipk2 (XM_037422147.1) was downregulated 233.0-fold in response to *B. bovis* infection and 183.0-fold in response to *B. bigemina* infection. Flavin-containing monooxygenases (FMOs), which may play a role in xenobiotic metabolism in insects [73], were differentially regulated by *Babesia* infection. *Rhipicephalus microplus* FMO5 (XM_037420399.1) downregulation was greater in response to *B. bovis* (56.0-fold) than to *B. bigemina* infection (14.0-fold). The decrease in metabolism in nonimmune tissues of infected insects was previously observed owing to a metabolic switch that redirects nutrients from nonimmune processes to immunity [71]. Also, the infected insects switched their metabolic organ, the fat body, from anabolism to the production of AMPs [71]. *Rhipicephalus microplus* hemocyte genes vitellogenin-2-like (XM_037432037.1) and vitellogenin-5-like (XM_037431896.1), which were found in the pathways of lipid transport and lipid localization (Table 8), were downregulated in response to *Babesia* infection (Table 7). Differentially regulated molecular function pathways and biological process pathways of lipid transport/lipid localization are shown in Figs. 12B and 13C, respectively. The differential regulation of lipid metabolism in *R. microplus* hemocytes may indicate a possible metabolic switch during the *Babesia* infection.

Along with differential regulation of metabolism, *Babesia* infection affected gene expression of the cytoskeleton constituents of *R. microplus* (Table 8). *Rhipicephalus microplus* spectrin beta chain-like (XM_037418224.1) was downregulated 19.0-fold during both *B. bovis* and *B. bigemina* infections. This gene belongs to the spectrin family of filamentous cytoskeletal proteins that stabilize plasma membranes and organize cell organelles [74]. *Anaplasma phagocytophilum* infection in *I. scapularis* downregulated the expression of the spectrin alpha chain in salivary glands to inhibit apoptosis and subvert host cell defenses [37]. The functional characterization of *R. microplus* spectrin beta chain will help us to understand how tick hemocytes respond to infection through the spectrin beta chain. The molecular pathways involving cytoskeleton protein binding, and its constituents, are shown in Fig. 12A and C.

### Hemocyte gene downregulation restricted to *B. bovis* infection

A total of 66 tick genes were downregulated in response to *B. bovis* infection only. Out of the 66 downregulated genes, 61 were identified as protein-coding genes, 2 were lncRNAs, and 3 were not found by the Ensemble annotation system [22]. Downregulation fold change of differentially regulated protein-coding hemocyte genes unique to *B. bovis* infection was between 3.0-fold and 164.0-fold, and in putative lncRNAs (XR_005110666.1 and XR_005110213.1) it was between 24.0-fold and 9.0-fold (Table 9). Primarily, cellular metabolism was differentially regulated through the downregulation of genes found in the cellular metabolic process (Table 10). Poly (ADP-ribose) polymerase tankyrase-like (XM_037418413.1) was substantially downregulated (165.0-fold) upon *B. bovis* infection. Tankyrase regulates Wnt signaling and glucose metabolism [75]. Wnt signaling regulates cell proliferation and survival by regulating the gene expression for cell differentiation [76]. The glucose metabolism fuels cell growth and cell division [77]. Downregulation of *R. microplus* poly (ADP-ribose) polymerase tankyrase may be linked to the survival mechanism of *B. bovis* by eliminating hemocytes upon the infection. Supporting this hypothesis, the transcription regulators, forkhead box protein P1-like (XM_037415909.1) and nuclear hormone receptor E75-like (XM_037427169.1), were also downregulated (9.0-fold and 9.0-fold, respectively) in response to *B. bovis* infection. Forkhead Box P1 (FOXP1) and nuclear hormone receptor E75 are shown to regulate cell differentiation, cell proliferation, and development [78, 79].

**Table 9:** Tick genes downregulated in response to *B. bovis* infection only with an FDR < 0.05

Accession no.	Gene	Fold decrease
XM_037418413.1	Rm poly [ADP-ribose] polymerase tankyrase-like	164.92
XM_037418490.1	Rm interferon regulatory factor 2-binding protein 1-like	59.80
XM_037425304.1	Rm E3 ubiquitin-protein ligase UBR5-like	27.35
XM_037425899.1	Rm oocyte zinc finger protein XICOF6-like	25.11
XR_005110213.1	Rm uncharacterized	23.98
XM_037418478.1	Rm mitogen-activated protein kinase-binding protein 1-like	16.75
XM_037435569.1	Rm glycogen-binding subunit 76A-like	13.40
XM_037424436.1	Rm uncharacterized	13.16
XM_037428234.1	Rm transcription factor SKN7-like	12.44
XM_037421326.1	Rm leukocyte elastase inhibitor-like	12.06
XM_037415340.1	Rm peregrin-like	11.83
XM_037429964.1	Rm probable ubiquitin carboxyl-terminal hydrolase FAF-X	11.62
XM_037417028.1	Rm hemocytin-like	10.08
XM_037413541.1	Rm glycine-rich cell wall structural protein 1-like	9.47
XM_037424958.1	Rm inactive serine protease PAMR1-like	9.37
XR_005110666.1	Rm uncharacterized	9.28
XM_037415909.1	Rm forkhead box protein P1-like	9.24
XM_037425985.1	Rm filamin-A-like	8.78
XM_037427169.1	Rm nuclear hormone receptor E75-like	8.69
XM_037430419.1	Rm GPI inositol-deacylase-like	8.59
XM_037432520.1	Rm deubiquitinating protein VCIP135-like	8.39
XM_037421190.1	Rm trypsin II-P29-like	8.14
XM_037417272.1	Rm apoptosis-stimulating of p53 protein 2-like	7.78
XM_037423849.1	Rm uncharacterized	7.63
XM_037431000.1	Rm uncharacterized	7.56
XM_037420425.1	Rm clotting factor B-like	7.46
XM_037426141.1	Rm IQ motif and SEC7 domain-containing protein 1-like	7.31
XM_037429845.1	Rm uncharacterized	6.77
XM_037431488.1	Rm hydroxyacid oxidase 1-like	6.71
XM_037418055.1	Rm protein numb-like	6.58
XM_037430565.1	Rm 17-beta-hydroxysteroid dehydrogenase type 6-like	6.53
XM_037432661.1	Rm RING finger and transmembrane domain-containing protein 2-like	6.48
XM_037424960.1	Rm inactive serine protease PAMR1-like	6.44
XM_037425973.1	Rm double-stranded RNA-binding protein Staufen homolog 2-like	5.99
XM_037432170.1	Rm phophastidylinositol 3-kinase regulatory subunit alpha-like	5.93
XM_037418573.1	Rm pancreatic lipase-related protein 2-like	5.86
XM_037430793.1	Rm myocardin-related transcription factor A-like	5.82
XM_037414003.1	Rm uncharacterized	5.78
XM_037431485.1	Rm hydroxyacid oxidase 1-like	5.76
XM_037431479.1	Rm hydroxyacid oxidase 1-like	5.75
XM_037413532.1	Rm acanthoscurrin-2-like	5.70
XM_037426084.1	Rm endosome/lysosome-associated apoptosis and autophagy regulator family member 2-like	5.69
XM_037422074.1	Rm cytochrome P450 1A1-like	5.67
XM_037426120.1	Rm phospholipase A and acyltransferase 3-like	5.61
XM_037413874.1	Rm uncharacterized	5.60
XM_037418350.1	Rm long-chain-fatty-acid–CoA ligase 3-like	5.50
XM_037434879.1	Rm peroxisomal (S)-2-hydroxy-acid oxidase GLO5-like	5.46
XM_037425091.1	Rm sulfotransferase 1C2-like	5.37
XM_037417100.1	Rm uncharacterized	4.91
XM_037416757.1	Rm uncharacterized	4.86
XM_037430521.1	Rm uncharacterized	4.82
XM_037423848.1	Rm uncharacterized	4.62
XM_037432104.1	Rm LIM domain and actin-binding protein 1-like	4.58
XM_037420162.1	Rm putative serine/threonine-protein kinase haspin homolog	4.43
XM_037416562.1	Rm glycine-rich cell wall structural protein-like	4.37
XM_037431726.1	Rm mitogen-activated protein kinase kinase kinase 1-like	4.18
XM_037413488.1	Rm serine protease 56-like	4.07
XM_037414892.1	Rm egalitarian protein homolog	4.04
XM_037421512.1	Rm uncharacterized	4.02
XM_037415919.1	Rm serine protease inhibitor swm-1-like	3.87
XM_037433675.1	Rm homer protein homolog 2-like	3.79
XM_037435563.1	Rm chorion peroxidase-like	3.78
XM_037421529.1	Rm glycoprotein 3-alpha-L-fucosyltransferase A-like	3.72
XM_037423124.1	Rm calsequestrin-2-like	3.70
XM_037425476.1	Rm transketolase-like	3.68
XM_037433402.1	Rm carboxypeptidase M-like	3.28

**Table 10:** Downregulated hemocyte genes following the *B. bovis* infection, grouped by biological process, molecular function, and cellular component categories defined by high-level GO terms

	N	High-level GO category	Genes
Biological process	19	GO:0044238 primary metabolic process GO:0071704 organic substance metabolic process	XM_037413488.1 XM_037414892.1 XM_037415909.1 XM_037417028.1 XM_037418413.1 XM_037418573.1 XM_037420425.1 XM_037421190.1 XM_037421326.1 XM_037421529.1 XM_037423848.1 XM_037423849.1 XM_037424958.1 XM_037427169.1 XM_037429964.1 XM_037431726.1 XM_037432520.1 XM_037432661.1 XM_037433402.1
	18	GO:0006807 nitrogen compound metabolic process	XM_037413488.1 XM_037414892.1 XM_037415909.1 XM_037417028.1 XM_037418413.1 XM_037420425.1 XM_037421190.1 XM_037421326.1 XM_037421529.1 XM_037423848.1 XM_037423849.1 XM_037424958.1 XM_037427169.1 XM_037429964.1 XM_037431726.1 XM_037432520.1 XM_037432661.1 XM_037433402.1
	13	GO:0044237 cellular metabolic process	XM_037414892.1 XM_037415909.1 XM_037417028.1 XM_037418413.1 XM_037420162.1 XM_037421326.1 XM_037421529.1 XM_037427169.1 XM_037429964.1 XM_037431726.1 XM_037432520.1 XM_037432661.1 XM_037433402.1
	11	GO:0006508 proteolysis	XM_037413488.1 XM_037417028.1 XM_037420425.1 XM_037421190.1 XM_037421326.1 XM_037423848.1 XM_037423849.1 XM_037424958.1 XM_037429964.1 XM_037432661.1 XM_037433402.1
	9	GO:0050789 regulation of biological process GO:0065007 biological regulation GO:0050794 regulation of cellular process	XM_037415909.1 XM_037417028.1 XM_037421326.1 XM_037426141.1 XM_037427169.1 XM_037430521.1 XM_037432170.1 XM_037432661.1 XM_037433675.1
	8	GO:0050896 response to stimulus	XM_037426141.1 XM_037427169.1 XM_037429845.1 XM_037430521.1 XM_037432170.1 XM_037432661.1 XM_037433675.1 XM_037435563.1
	7	GO:0051716 cellular response to stimulus	XM_037426141.1 XM_037427169.1 XM_037430521.1 XM_037432170.1 XM_037432661.1 XM_037433675.1 XM_037435563.1
	5	GO:0023052 signaling	XM_037426141.1 XM_037427169.1 XM_037430521.1 XM_037432170.1 XM_037433675.1
	5	GO:0019222 regulation of metabolic process	XM_037415909.1 XM_037417028.1 XM_037421326.1 XM_037427169.1 XM_037432661.1
	3	GO:0009058 biosynthetic process	XM_037415909.1 XM_037421529.1 XM_037427169.1
	2	GO:0048519 negative regulation of biological process	XM_037417028.1 XM_037421326.1
	2	GO:0006950 response to stress GO:0042221 response to chemical	XM_037432661.1 XM_037435563.1
	2	GO:0009056 catabolic process	XM_037429964.1 XM_037432661.1
	2	GO:0044419 biological process involved in interspecies interaction between organisms	XM_037417272.1 XM_037429845.1
	2	GO:0048583 regulation of response to stimulus	XM_037426141.1 XM_037432661.1
	2	GO:0065009 regulation of molecular function	XM_037417028.1 XM_037421326.1
	1	GO:0032501 multicellular organismal process GO:0032502 developmental process GO:0048856 anatomical structure development	XM_037417028.1
	1	GO:0048518 positive regulation of biological process GO:0009893 positive regulation of metabolic process	XM_037432661.1
	1	GO:0051179 localization GO:0035821 modulation of process of another organism GO:0051234 establishment of localization	XM_037417272.1
	1	GO:0071840 cellular component organization or biogenesis GO:0016043 cellular component organization	XM_037425985.1
	1	GO:0009605 response to external stimulus GO:0009607 response to biotic stimulus GO:0051707 response to another organism	XM_037429845.1
	1	GO:0023051 regulation of signaling	XM_037426141.1
Molecular function	12	GO:0005515 protein binding	XM_037415340.1 XM_037417028.1 XM_037417272.1 XM_037418413.1 XM_037418478.1 XM_037425304.1 XM_037425985.1 XM_037426141.1 XM_037432104.1 XM_037432170.1 XM_037433675.1 XM_037435569.1
	12	GO:0016787 hydrolase activity GO:0140096 catalytic activity acting on a protein	XM_037413488.1 XM_037414892.1 XM_037418573.1 XM_037420425.1 XM_037421190.1 XM_037423848.1 XM_037423849.1 XM_037424958.1 XM_037429964.1 XM_037430419.1 XM_037432520.1 XM_037433402.1
	10	GO:0043167 ion binding	XM_037422074.1 XM_037423124.1 XM_037425304.1 XM_037425476.1 XM_037427169.1 XM_037431479.1 XM_037431726.1 XM_037432104.1 XM_037432661.1 XM_037433402.1
	9	GO:0097159 organic cyclic compound binding GO:1901363 heterocyclic compound binding	XM_037414892.1 XM_037415909.1 XM_037422074.1 XM_037425304.1 XM_037425973.1 XM_037427169.1 XM_037431479.1 XM_037431726.1 XM_037435563.1
	9	GO:0008233 peptidase activity	XM_037413488.1 XM_037420425.1 XM_037421190.1 XM_037423848.1 XM_037423849.1 XM_037424958.1 XM_037429964.1 XM_037432520.1 XM_037433402.1
	8	GO:0016740 transferase activity	XM_037418413.1 XM_037420162.1 XM_037421529.1 XM_037425091.1 XM_037425304.1 XM_037425476.1 XM_037431726.1 XM_037432661.1
	6	GO:0004252 serine-type endopeptidase activity GO:0008236 serine-type peptidase activity GO:0017171 serine hydrolase activity	XM_037413488.1 XM_037420425.1 XM_037421190.1 XM_037423848.1 XM_037423849.1 XM_037424958.1
	5	GO:0016491 oxidoreductase activity	XM_037422074.1 XM_037430565.1 XM_037431479.1 XM_037434879.1 XM_037435563.1
	3	GO:0060089 molecular transducer activity GO:0038023 signaling receptor activity	XM_037417100.1 XM_037427169.1 XM_037430521.1
	3	GO:0098772 molecular function regulator activity GO:0030234 enzyme regulator activity	XM_037417028.1 XM_037421326.1 XM_037426141.1
	3	GO:0097367 carbohydrate derivative binding	XM_037417028.1 XM_037431479.1 XM_037431726.1
	2	GO:0003700 DNA-binding transcription factor activity	XM_037415909.1 XM_037427169.1
	2	GO:0036094 small molecule binding	XM_037431479.1 XM_037431726.1
	2	GO:0051015 actin filament binding GO:0044877 protein-containing complex binding GO:0003779 actin binding	XM_037425985.1 XM_037432104.1
	2	GO:0004843 cysteine-type deubiquitinase activity GO:0101005 deubiquitinase activity GO:0008234 cysteine-type peptidase activity	XM_037429964.1 XM_037432520.1
	2	GO:0004842 ubiquitin-protein transferase activity	XM_037425304.1 XM_037432661.1
	1	GO:0004181 metallocarboxypeptidase activity	XM_037433402.1
	1	GO:0016209 antioxidant activity GO:0004601 peroxidase activity	XM_037435563.1
	1	GO:0004879 nuclear receptor activity GO:0098531 ligand-activated transcription factor activity	XM_037427169.1
	1	GO:0005085 guanyl-nucleotide exchange factor activity	XM_037426141.1
	1	GO:0043176 amine binding	XM_037429845.1
	1	GO:0003950 NAD + ADP-ribosyltransferase activity	XM_037418413.1
Cellular component	6	GO:0016020 membrane	XM_037417100.1 XM_037417272.1 XM_037421529.1 XM_037429845.1 XM_037430521.1 XM_037432661.1
	3	GO:0005576 extracellular region	XM_037417028.1 XM_037421326.1 XM_037423848.1
	2	GO:0043226 organelle GO:0043227 membrane-bounded organelle	XM_037418413.1 XM_037427169.1
	1	GO:0032991 protein-containing complex	XM_037414892.1
	1	GO:0005615 extracellular space	XM_037421326.1

N: the number of upregulated hemocyte genes that fall under a similar GO term

**Fig. 14:**
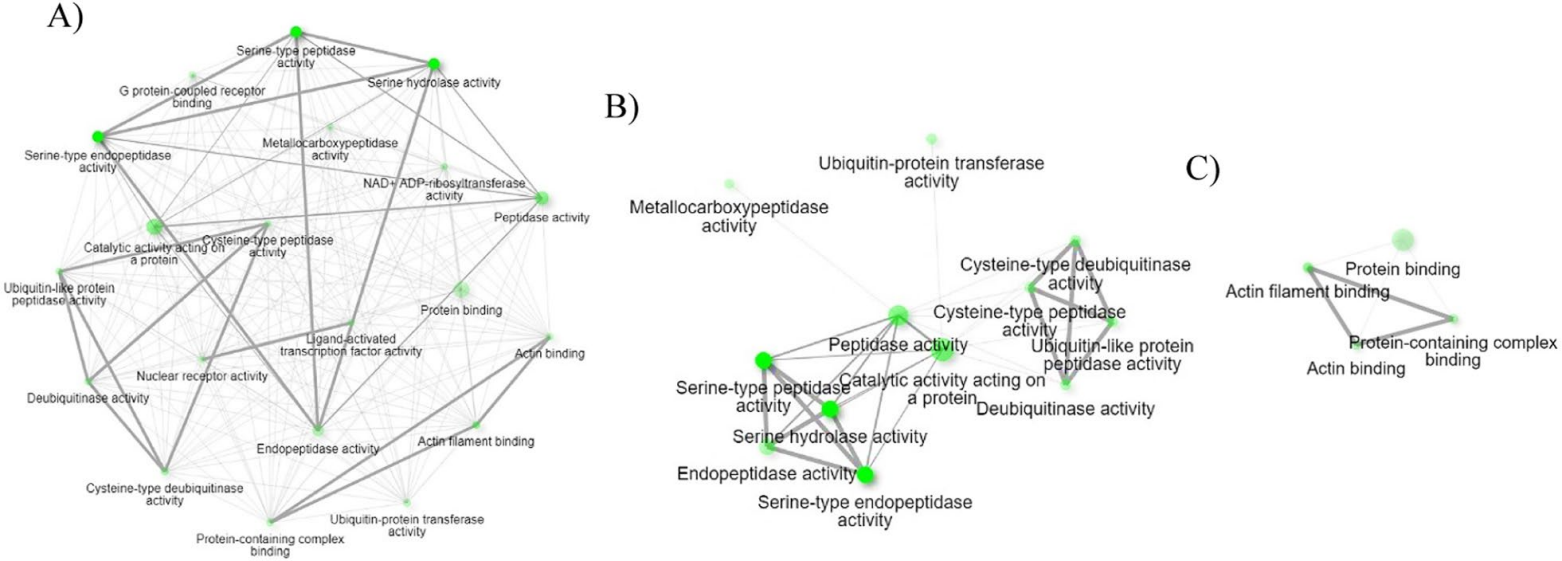
Molecular functional pathways related to downregulated hemocyte genes following *B. bovis* infection. **A** Network of all the molecular functional pathways, **B** molecular functional pathways involved in peptidase activity, and **C** protein binding. Each node of the network represents an enriched GO term and the related GO terms are connected by a line. The thickness of the line reflects the percent of overlapping genes, and the size of the node corresponds to the number of genes

Moreover, several hemocyte genes of protein binding pathways (Table 10) linked to cell survival are downregulated after *B. bovis* infection. *Rhipicephalus microplus* mitogen-activated protein kinase-binding protein 1-like was downregulated 17.0-fold. Mitogen-activated protein kinases (MAPKs) are a group of proteins that play key roles in cell proliferation, cell differentiation, transcription regulation, and development [80]. *Rhipicephalus microplus* E3 ubiquitin-protein ligase UBR5-like (XM_037425304.1) was downregulated upon the *B. bovis* infection (27.0-fold), and E3 ubiquitin-protein ligase is reported to be required for protein quality control pathways in the cytoplasm and nucleus of cells [81]. *Rhipicephalus microplus* peregrin-like (XM_037415340.1), which regulates chromatin and plays a role in epigenetic signaling [82], was downregulated 12.0-fold. *Rhipicephalus microplus* filamin-A-like (XM_037425985.1), which is related to cellular component organization or biogenesis pathways of hemocytes, was differentially expressed upon *B. bovis* infection by a 9.0-fold decrease (Table 10). *Drosophila* filamin is reported to organize actin filaments during oocyte development [83]. The network of actin binding associated with downregulated genes in response to *B. bovis* infection is shown in Fig. 14A, C. Further, the glycogen biosynthesis is differentially regulated upon *B. bovis* infection via the downregulation of glycogen-binding subunit 76A. This gene is predicted to regulate the process of glycogen biosynthesis in fruit flies [84]. Therefore, the process of glucose fueling to the cell may have been affected, resulting in halted cell growth and cell division upon *B. bovis* infection. *Rhipicephalus microplus* glycogen-binding subunit 76A-like (XM_037435569.1) was decreased 13.4-fold. Downregulated hemocyte genes associated with cell growth and survival may benefit *B. bovis* parasites by helping them evade hemocytes.

*R. microplus* hemocytes differentially regulated the components of the innate immune response, including serine proteases, in response to *B. bovis* infection (Table 10). Serine proteases are one of the key regulators of the innate immune response of insects and are crucial coagulation cascade components, which include clotting factor C, B, and pro-clotting enzymes [85, 86]. Serine proteases are involved in synthesis of AMPs, which directly kill invading pathogens and are involved in protein degradation for the controlled breakdown of immune complexes [87]. This RNA-Seq study reports downregulation of several serine-type endopeptidases (Table 10), including inactive serine protease PAMR1-like (XM_037424958.1), trypsin II-P29-like (XM_037421190.1), clotting factor B-like (XM_037420425.1), and serine protease 56-like (XM_037413488.1). In addition, peptidases including probable ubiquitin carboxyl-terminal hydrolase FAF-X (XM_037429964.1) and deubiquitinating protein VCIP135-like (XM_037432520.1) were downregulated 9.0-, 8.0-, 7.0-, 4.0-, 12.0-, and 8.0-fold, respectively. Together, several genes involved in proteolysis pathways (Table 10) were differentially regulated following *B. bovis* infection, including a 12.0-fold decrease of leukocyte elastase inhibitor-like (XM_037421326.1) and a 7.0-fold decrease of hydroxyacid oxidase 1-like (XM_037431488.1). The network of pathways involving molecular functions of peptidases found among downregulated genes in response to *B. bovis* infection are illustrated in Fig. 14A and B.

The survival mechanism of *B. bovis* in tick hemolymph is unknown. It could be a common mechanism that is inherent to protozoan [62] or a *B. bovis*-specific response to the tick hemocyte defense. This study reported the differential regulation of *R. microplus* hemocytin-like (XM_037417028.1), which was downregulated 10.0-fold. Hemocytin is a lectin released from granulocytes in response to invading microbes and plays a major role in insect coagulation, nodulation, and encapsulation [68, 69]. *Nosema bombycis* infection in silkworms results in its hemocytin binding to the pathogen leading to hemocyte aggregation and melanization [68]. Differential regulation of *R. microplus* hemocytin upon *B. bovis* infection indicates a control mechanism involving hemocytin. This RNA-Seq study also reported two genes that were found in the biological process pathway involved in interspecies interaction between organisms (Table 10). Apoptosis-stimulating p53 protein 2-like (XM_037417272.1) and uncharacterized gene XM_037429845.1 were downregulated 8.0-fold and 7.0-fold, respectively, and these two genes were identified in the hemocyte membrane (Table 10). Particularly, *R. microplus* apoptosis-stimulating p53 protein 2 was found in the biological process pathway for the establishment of localization and modulation of the process of another organism and the uncharacterized gene XM_037429845.1 was found in the biological pathway response to another organism (Table 10). Previous studies have indicated that the interaction of bacterial pathogens and host cells induces DNA damage that interrupts cellular homeostasis [88]. Protein p53 prevents damaged DNA from replicating by causing cell cycle arrest and triggering apoptosis, which removes damaged cells. Previously it was revealed that p53 plays a role in controlling bacterial infection. Certain bacteria inhibit p53 through mechanisms, including protein degradation, transcriptional inhibition, and posttranslational modifications, and therefore, it was suggested that the inhibition of p53 may confer successful infection [88]. *Rhipicephalus microplus* p53 protein found in this study and the uncharacterized gene XM_037429845.1 may be involved in cellular stress in response to the interaction between *B. bovis* parasites and hemocytes. Investigating this probable stress response mechanism unique to *B. bovis* infection will define insightful information on vector-pathogen interaction.

### Hemocyte gene downregulation restricted to *B. bigemina* infection

**Fig. 15:**
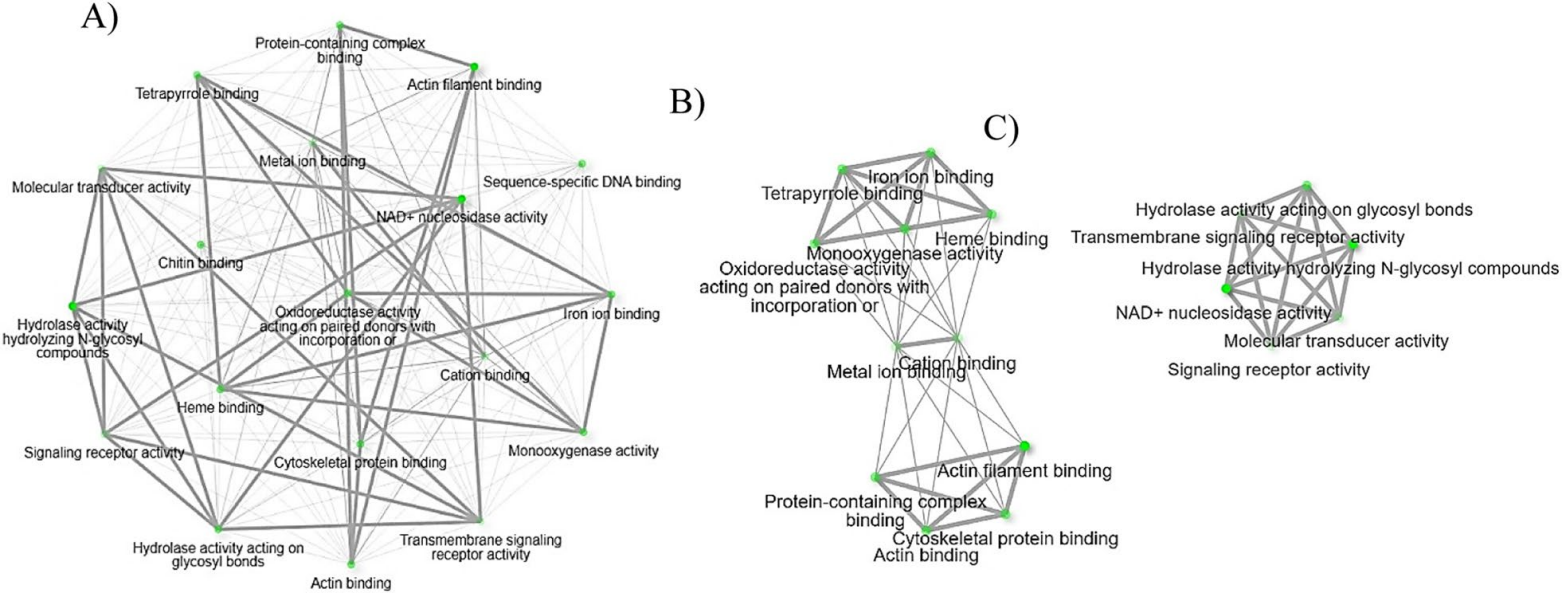
Molecular functional pathways related to downregulated hemocyte genes following *B. bigemina* infection. **A** Network of all the molecular functional pathways, **B** molecular functional pathways involved in iron binding, and **C** hydrolase activity. Each node of the network represents an enriched GO term and the related GO terms are connected by a line. The thickness of the line reflects the percent of overlapping genes, and the size of the node corresponds to the number of genes

There were nine hemocyte genes downregulated in response to *B. bigemina* infection only. Out of nine downregulated genes, seven were identified as protein-coding genes, and two were lncRNAs, on the basis of the Ensemble annotation system [22]. Suppressed gene expressions unique to *B. bigemina* infection were found to range from a 5.0-fold to a 306.0-fold decrease for protein-coding genes and from a 155.0-fold to a 16.0-fold decrease for putative lncRNAs (XR_005110200.1 and XR_005109610.1) (Table 11). Substantial downregulation (306.0-fold) was detected in the expression of the cytoskeletal gene, LIM domain, and actin-binding protein 1-like/transcript variant X6 (XM_037432102.1) that is found in the molecular functional pathways of cytoskeletal protein binding/actin filament binding (Table 12). The host cytoskeletal network is known to be encountered by intracellular bacteria for adherence and invasion using actin and related proteins to establish infection [89]. Rearrangement of the cytoskeleton was previously recognized in the *I. scapularis* response to intracellular bacterium *A. phagocytophilum*, where the ratio of monomeric to filamentous actin was altered [38]. Along with the *R. micoplus* actin-binding protein, this study reports the downregulation of collagen alpha-1(III) chain-like (XM_037424593.1) found in the chitin-binding pathway and collagen trimer (Table 12). Chitin forms the exoskeletal framework, which provides support to actin-based muscle contraction [90]. The downregulation of *R. microplus* collagen alpha-1(III) chain (87.0-fold) indicates probable changes in cytoskeletal components unique to *B. bigemina* infection. The network of actin filament binding pathways involving *R. microplus* LIM domain, and actin-binding protein 1-like/transcript variant X6 upon *B. bigemina* infection is shown in Fig. 15A and B.

**Table 11:** Tick genes downregulated in response to *B. bigemina* infection only with an FDR < 0.05

Accession no.	Gene	Fold decrease
XM_037432102.1	Rm LIM domain and actin-binding protein 1-like/transcript variant X6	306.29
XR_005110200.1	Rm uncharacterized	155.36
XM_037424593.1	Rm collagen alpha-1(III) chain-like	86.95
XM_037429085.1	Rm piggyBac transposable element-derived protein 3-like	18.97
XM_037423917.1	Rm cytochrome P450 3A24-like	16.95
XR_005109610.1	Rm uncharacterized	15.85
XM_037418715.1	Rm leucine-rich repeat-containing protein 15-like	12.48
XM_037418711.1	Rm protein toll-like	12.19
XM_037420306.1	Rm uncharacterized	5.75

**Table 12:** Downregulated hemocyte genes following the *B. bigemina* infection, grouped by biological process, molecular function, and cellular component categories defined by high-level GO terms

	N	High-level GO category	Genes
Biological process	1	GO:0002221 pattern recognition receptor signaling pathway GO:0002224 toll-like receptor signaling pathway GO:0002764 immune response-regulating signaling pathway GO:0002682 regulation of immune system process GO:0050776 regulation of immune response GO:0002376 immune system process GO:0006955 immune response GO:0048583 regulation of response to stimulus	XM_037418711.1
Molecular function	1	GO:0003953 NAD + nucleosidase activity GO:0016799 hydrolase activity hydrolyzing N-glycosyl compounds GO:0016798 hydrolase activity acting on glycosyl bonds GO:0038023 signaling receptor activity GO:0060089 molecular transducer activity GO:0004888 transmembrane signaling receptor activity	XM_037418711.1
	1	GO:0051015 actin filament binding GO:0003779 actin binding GO:0008092 cytoskeletal protein binding GO:0044877 protein-containing complex binding	XM_037432102.1
	1	GO:0004497 monooxygenase activity GO:0005506 iron ion binding GO:0020037 heme binding GO:0016705 oxidoreductase activity acting on paired donors with incorporation or reduction of molecular oxygen GO:0046906 tetrapyrrole binding	XM_037423917.1
		GO:0008061 chitin binding	XM_037424593.1
		GO:0043565 sequence-specific DNA binding	XM_037420306.1
Cellular component	1	GO:0005581 collagen trimer	XM_037424593.1

N: the number of upregulated hemocyte genes that fall under a similar GO term

**Fig. 16:**
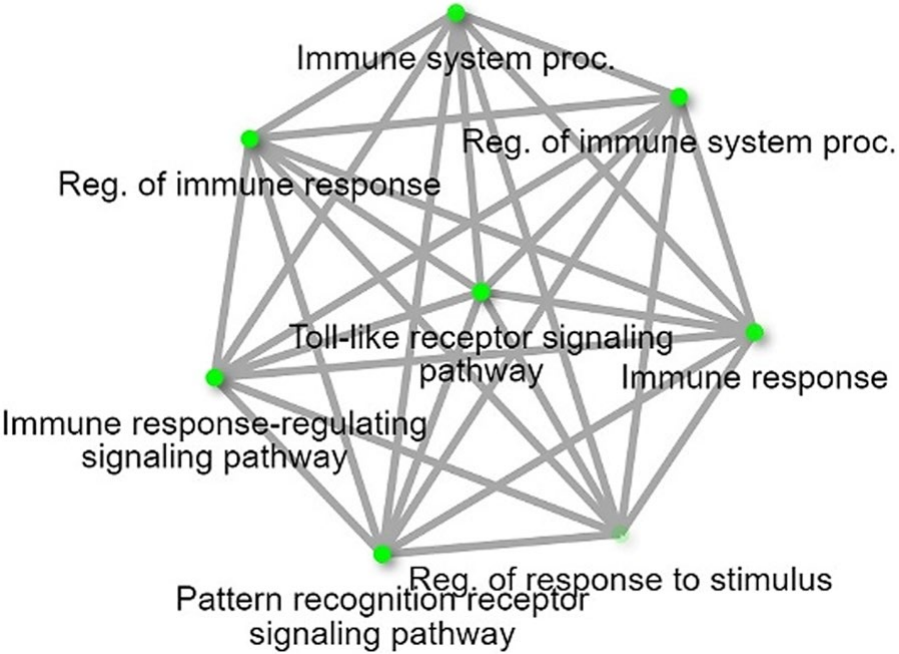
Network of biological process pathways related to downregulated hemocyte genes following *B. bigemina* infection. Each node of the network represents an enriched GO term and the related GO terms are connected by a line. The thickness of the line reflects the percent of overlapping genes, and the size of the node corresponds to the number of genes

The only innate immune signaling pathway that is differentially regulated during *B. bigemina* infection is the toll-like receptor (TLR) signaling pathway or pattern recognition receptor signaling pathway (Table 12). *Rhipicephalus microplus* protein toll-like (XM_037418711.1) was downregulated 12.0-fold following infection only by*B. bigemina*. The TLR pathway of the model insect *Drosophila* recognizes and eliminates fungi and gram-positive bacteria by binding to pathogen-associated molecular patterns. Also, the TLR pathway regulates the synthesis of AMPs that are secreted into the hemolymph [91, 92]. Previous studies have suggested that *A. marginale* regulates the TLR pathway by downregulating the gene expression of the toll pathway for their successful infection in ticks [70]. This study additionally found the downregulation of protein toll-like (XM_037418714.1) 16.0-fold and leucine-rich repeat-containing protein 15-like (XM_037418712.1) 12.0-fold during *B. bovis* infection or *B. bigemina* infection (Table 11). In insects, leucine-rich repeat-containing toll-proteins act as pattern recognition receptors that can bind to pathogen-associated molecular patterns [93]. Thereby, findings in this RNA-Seq study suggest that *B. bigemina* kinete-stage parasites may regulate the TLR pathway for its successful establishment in hemolymph, thereby defeating hemocytes. The network of immune signaling pathways detected among downregulated genes that are unique to *B. bigemina* infection are illustrated in Figs. 15C and 16.

Along with the regulation of immune pathways, infection in ticks often affects their metabolism, resulting in the loss of tick fitness [94]. *Babesia bigemina* infection differentially regulated the expression of cytochrome P450 3A24-like (XM_037423917.1). *Rhipicephalus. microplus* cytochrome P450 3A24 was downregulated 17.0-fold, and it was identified in the heme binding pathway (Table 12). Cytochrome P450 is a heme-dependent enzyme that catalyzes oxidative reactions [95]. Ticks lack enzymes for heme breakdown, thereby storing exogenous heme by heme-binding proteins [97]. Heme is essential for the reproduction and development of ticks [97], and the differential regulation of heme binding upon *B. bigemina* infection may suggest the possible mechanism that causes the loss of tick fitness in infected ticks. The network of molecular pathways involving heme binding is illustrated in Fig. 15B.

## Conclusions

This study unveils the dynamic transcriptome of hemocytes in the cattle tick, *R. microplus*, when challenged with *B. bovis* or *B. bigemina*. These data significantly expand our understanding of the potential hemocyte genes involved in combating infections and influencing the development of *Babesia* parasites within tick vectors. Upon *Babesia* kinete infection in the tick hemolymph, the hemocytes of *R. microplus* robustly regulated genes associated with cell metabolism, cytoskeletal rearrangement, the tick’s innate immune response, cell growth, and cell survival. Notably, genes related to cell metabolism, endopeptidase inhibitors, and AMPs were commonly upregulated in response to both *Babesia* parasites. Additionally, *B. bovis* infection triggered the upregulation of genes linked to cytoskeletal rearrangement, pathogen-associated molecular patterns, and the production of eicosanoids, showcasing the hemocytes’ response specific to *B. bovis* parasites. Equally, hemocytes exhibited a*B. bigemina*-specific response through the substantial upregulation of salivary cystatin and *B. bigemina*-specific AMPs, suggesting the underlying mechanism of hemocytes toward *B. bigemina* parasites. Conversely, downregulated genes were primarily associated with hematopoiesis, cell growth, and cell survival, indicating the regulation of the hemocyte population in response to *Babesia* infection. While this research offers valuable insights, it also contains limitations. Specifically, the study does not include information on how low levels of *Babesia* parasitemia affect gene regulation in tick hemocytes. Previous studies have shown that female ticks fed on low levels of *B. bovis* parasitemia exhibit reduced levels of kinetes in their hemolymph [97]. Another limitation is that this study employed bulk RNA-Seq, which does not capture the responses of individual hemocyte cells against *Babesia* parasites. Further characterization of *B. bovis* or *B. bigemina*-specific hemocyte genes is necessary to identify gene markers that can enhance our understanding of tick infection dynamics and aid in the development of targeted treatments. Nevertheless, this RNA-Seq study provides an important framework for understanding tick hemocyte responses to *Babesia* infection, shedding light on the adaptive mechanisms involved in the *Babesia*–tick interaction.

## Supplementary Information

Additional File 1: Primers used for the PCR reactions

Additional File 2: Differentially expressed genes with their corresponding FDR values

Additional File 3: Differentially regulated protein coding genes positioned in *R. microplus* chromosomes

Additional File 4: Additional genes validated by qRT-PCR

## Data Availability

All raw and processed RNA-Seq data generated in this study were submitted into the Gene Expression Omnibus database under accession no. GSE243493 and are available at the following URL: https://www.ncbi.nlm.nih.gov/geo/query/acc.cgi?acc=GSE243493.

## Abbreviations

PCR: Polymerase chain reaction
qRT-PCR: Quantitative reverse transcription-polymerase chain reaction
cDNA: Complementary deoxyribonucleic acid
USDA-ARS: United States Department of Agriculture-Agricultural Research Service
DNase: Deoxyribonuclease
mRNA: Messenger ribonucleic acid
BLAST: Basic Local Alignment Search Tool
NCBI: National Center for Biotechnology Information
