# Supplementary material for: Transcriptomic analysis of Rhipicephalus microplus hemocytes from female ticks infected with Babesia bovis or Babesia bigemina
Source: Parasit Vectors. 2025 Feb 3;18:37. doi: 10.1186/s13071-025-06662-w (PMC11789329; doi:10.1186/s13071-025-06662-w)
Supplement: Supplementary file 1 — Additional File 1: Primers used for the PCR reactions [file 13071_2025_6662_MOESM1_ESM.docx]

**Additional Table 1.** Primers used for the PCR reactions.

| Gene | Primer sequence | Tm  (^o^C) | Product size | Primer efficiency (%) |
| --- | --- | --- | --- | --- |
| Microplusin-like (XM_037413835.1) | F-CACCATCACCACCACTGAAA | 60 | 98 bp | 91.6 |
|  | R-GATTAAGCACGTTGCAGCAG |  |  |  |
| Defensin-like (XM_037414789.1) | F-GTGCTTGTTGCTGGACTGAT | 60 | 99 bp | 112.6 |
|  | R-GAATACAGGGCAACCGAAAC |  |  |  |
| Homeodomain-interacting protein kinase 2-like (XM_037422147.1) | F-GTGGCTCCCAACACATCTAAA | 60 | 108 bp | 91.1 |
|  | R-CAGAAACTCCAGCACCTCATAC |  |  |  |
| Protein toll-like (XM_037418714.1) | F-GGCGTACGTTCGTGTTACAA | 60 | 96 bp | 101.7 |
|  | R-TTCCTCGTGGTGTCCTTTCT |  |  |  |
| Terminal nucleotidyltransferase 5C-like (XM_037426165.1) | F-TTGCACAAGCTCGTCAAGGT | 60 | 92 bp | 106.8 |
|  | R-AACTTGAGCTCCACGTTTCG |  |  |  |
| Keratin-associated protein 19-2-like (XM_037414510.1) | F-GCGCTGGTCTTGAAAACTTC | 60 | 115 bp | 93.2 |
|  | R-CTGTAACCACGGCTTCCTTT |  |  |  |
| Ixodidin-like (XM_037429385.1) | F-TCGAGCAGCAACTTGAGAGA | 60 | 115 bp | 92.0 |
|  | R-GGACATAAGCTTCCGCATTC |  |  |  |
| Acanthoscurrin-2-like XM_037413526.1 | F-CGGATTCGGACAGTCTTCTT | 60 | 99 bp | 100.7 |
|  | R-CTGCTGCACGAACTCTTTTG |  |  |  |
| 40S ribosomal protein S3a (XM_037430639.1) | F-CAGGAACATTGGCAAGACCT | 60 | 94 bp | 100.3 |
|  | R-GGCTTGGGAGACTTCAAACA |  |  |  |
| Ribosomal protein L4 (CV447629.1) | F-AGGTTCCCCTGGTGGTGAG | 60 | 149 bp | 97.1 |
|  | R-GTTCCTCATCTTTCCCTTGCC |  |  |  |
| Glyceraldehyde-3-phosphate dehydrogenase (CK180824) | F-AGTCCACCGGC GTCTTCCTCA | 60 | 124 bp | 92.4 |
|  | R-GTGTGGTTCACA CCCATCACAA |  |  |  |
| *B. bovis* kinete stage specific gene (BBOV_I002220) | F-GGGCAATGTTAATGGCAAGATAG | 60 | 116 bp | 102.0 |
|  | R-CCTCAACCTCAGCCTCAATAAG |  |  |  |
| *B. bigemina* kinete stage specific gene (BBOND_0206730) | F-GCTGATGGCACCAAAGAGTT | 60 | 109 bp | 100.4 |
|  | R-CGAACGCATCCTTAACCATC |  |  |  |
| **B. bovis* kinete stage specific gene (BBOV_I002220)-plasmid | F–GGGCAATGTTAATGGCAAGATAG | 60 | 116 bp | N/A |
|  | R–CCTCAACCTCAGCCTCAATAAG |  |  |  |
| **B. bigemina* kinete stage specific gene (BBOND_0206730)-plasmid | F-ATGAGCACCAATCACGACAG | 60 | 590 bp | N/A |
|  | R-AGGGCACCAGGTAACACAAA |  |  |  |
| *Calreticulin (AY395254.1) | F-TCGTGGCGTTTGATGATG | 60 | 118 bp | N/A |
|  | R-CGAACTTTCCGAGGTTGTC |  |  |  |

N/A= Not Applicable. *The primers used for the conventional PCR.
