# Supplementary material for: Transcriptomic analysis of Rhipicephalus microplus hemocytes from female ticks infected with Babesia bovis or Babesia bigemina
Source: Parasit Vectors. 2025 Feb 3;18:37. doi: 10.1186/s13071-025-06662-w (PMC11789329; doi:10.1186/s13071-025-06662-w)
Supplement: Supplementary file 3 — Additional File 3: Differentially regulated protein coding genes positioned in R. microplus chromosomes [file 13071_2025_6662_MOESM3_ESM.docx]

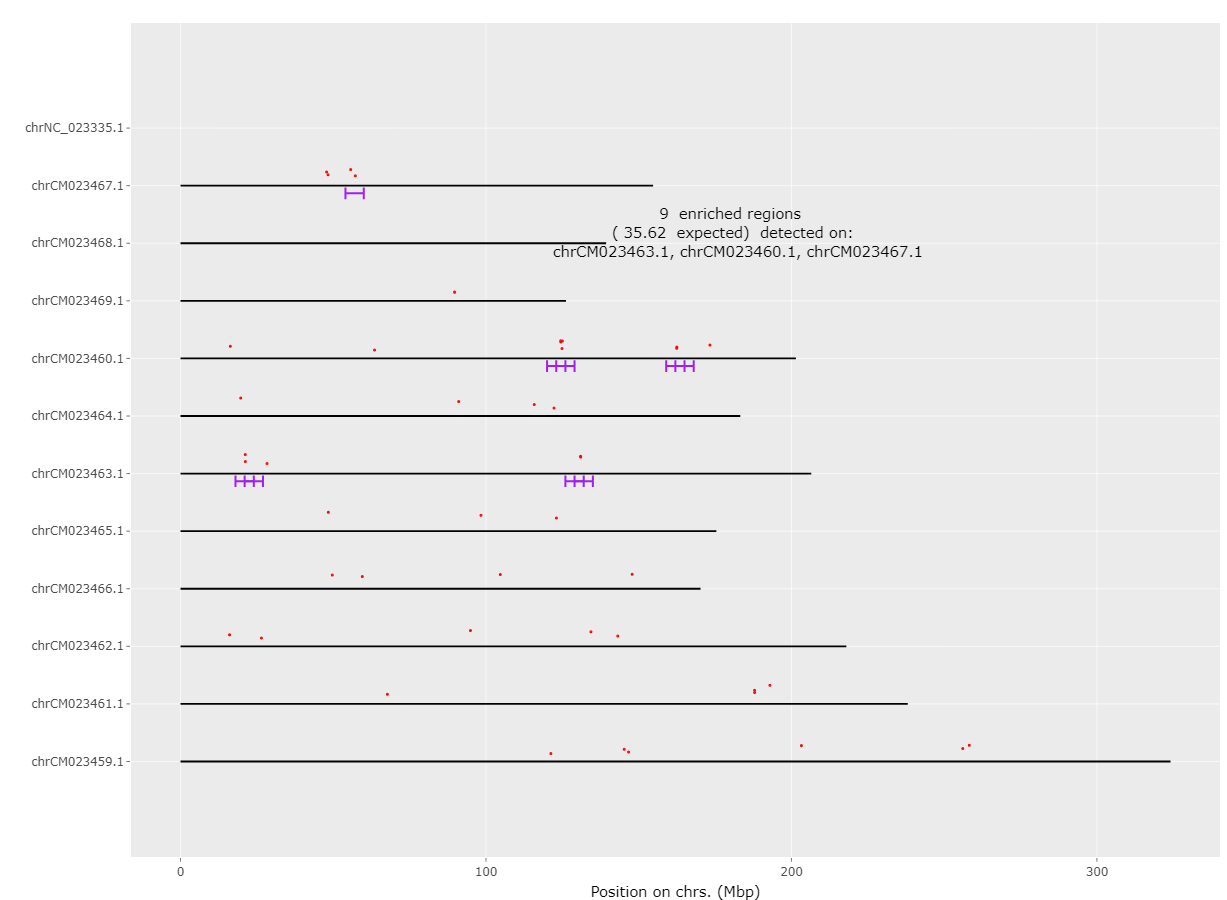


**Additional Fig. 2** Upregulated protein-coding genes following *Babesia* infection are positioned on *R. microplus* chromosomes (ShinyGO 0.80). Genes are represented by red dots and the purple lines indicate regions where these genes are statistically enriched, compared to the density of genes in the background. FDR<0.05.


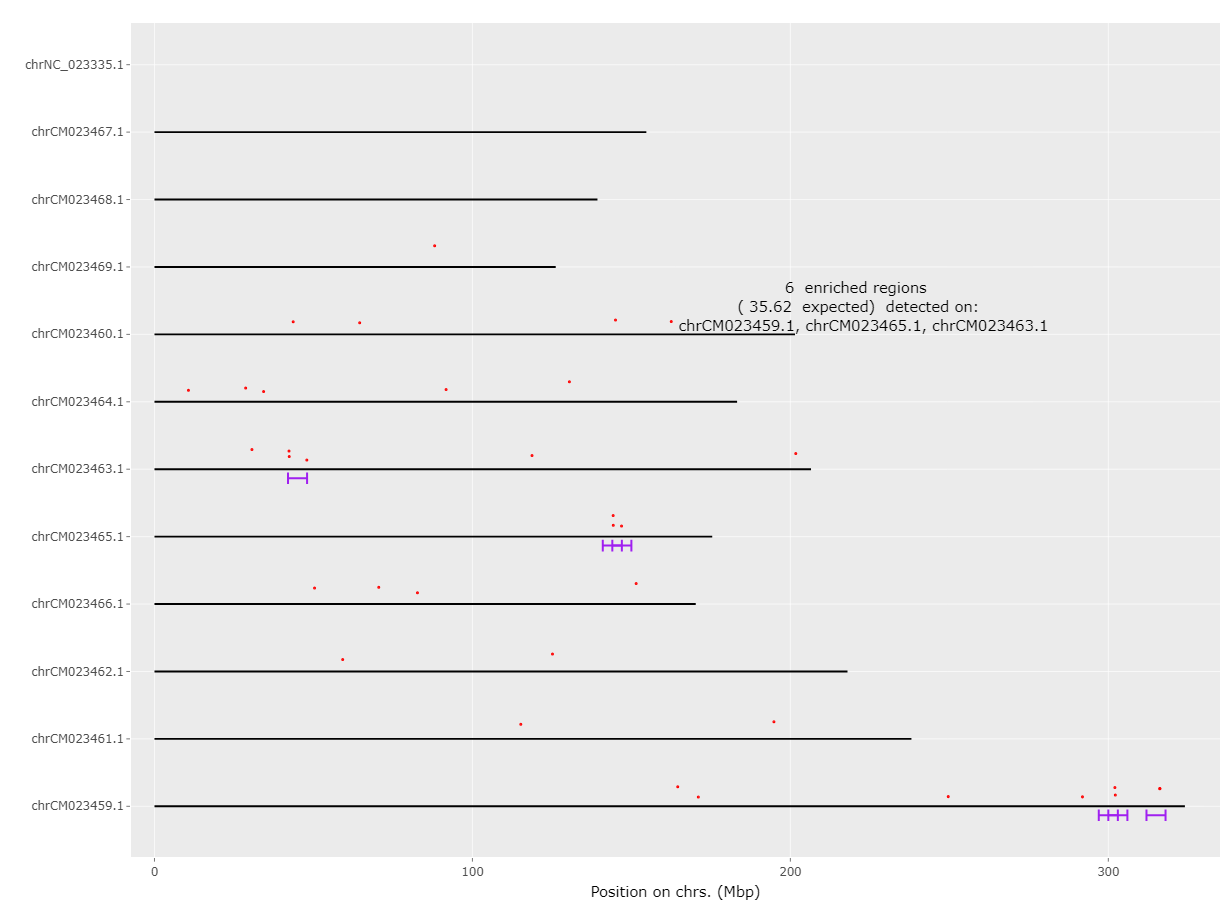


**Additional Fig. 3** Upregulated protein-coding genes restricted to *B. bovis* infection are positioned on *R. microplus* chromosomes (ShinyGO 0.80). Genes are represented by red dots and the purple lines indicate regions where these genes are statistically enriched, compared to the density of genes in the background. FDR<0.05.


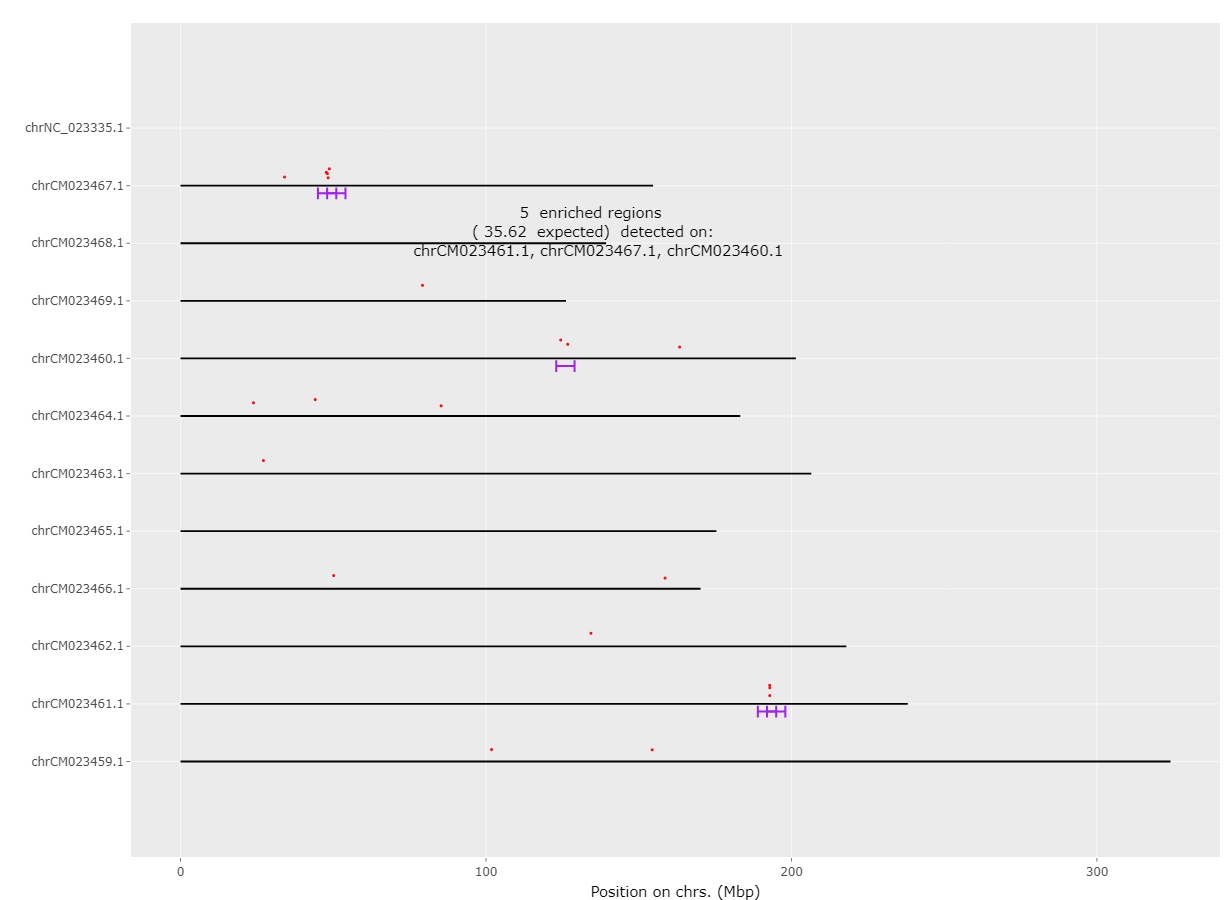


**Additional Fig. 4** Upregulated protein-coding restricted to *B. bigemina* infection are positioned on *R. microplus* chromosomes (ShinyGO 0.80). Genes are represented by red dots and the purple lines indicate regions where these genes are statistically enriched, compared to the density of genes in the background. FDR<0.05.


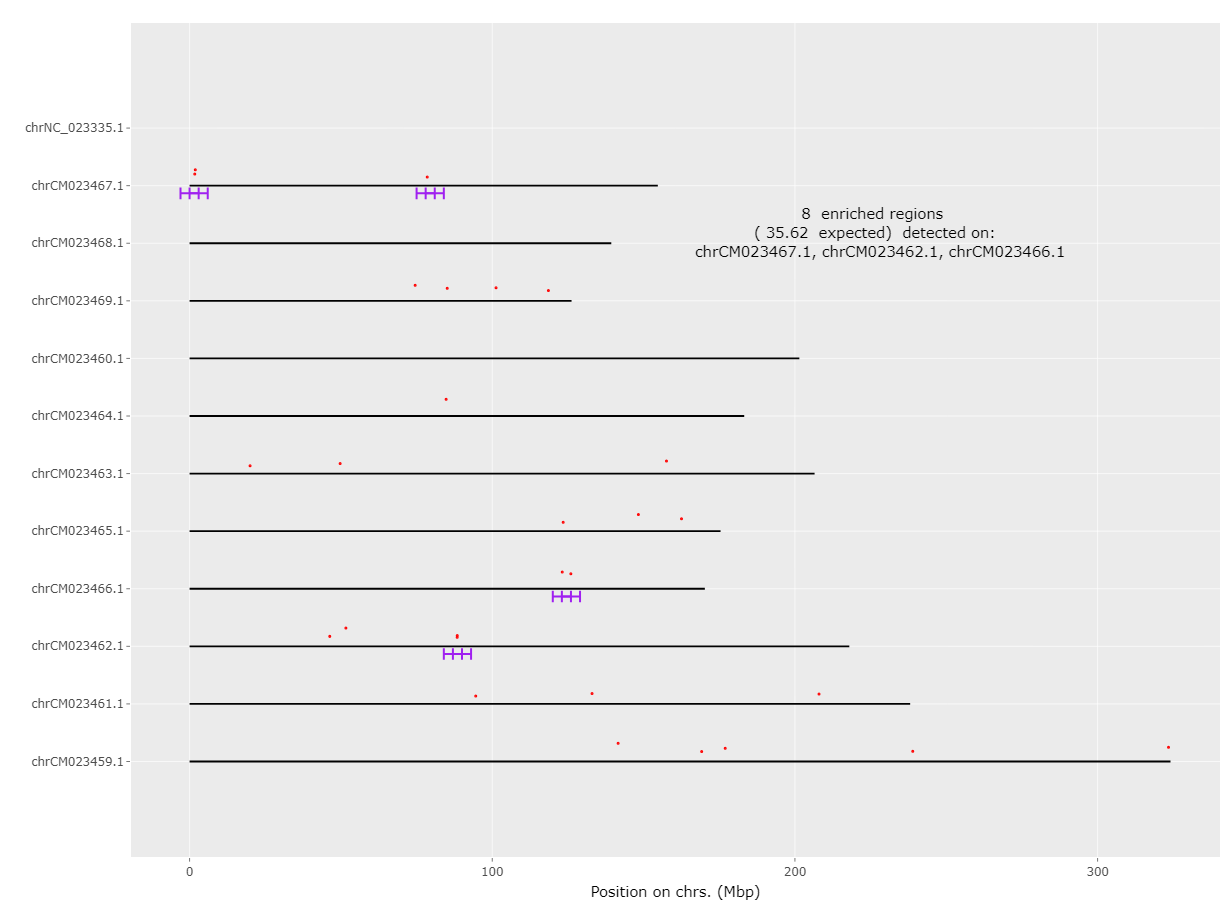


**Additional Fig. 5** Downregulated protein-coding genes following *Babesia* infection are positioned on *R. microplus* chromosomes (ShinyGO 0.80). Genes are represented by red dots and the purple lines indicate regions where these genes are statistically enriched, compared to the density of genes in the background. FDR<0.05.


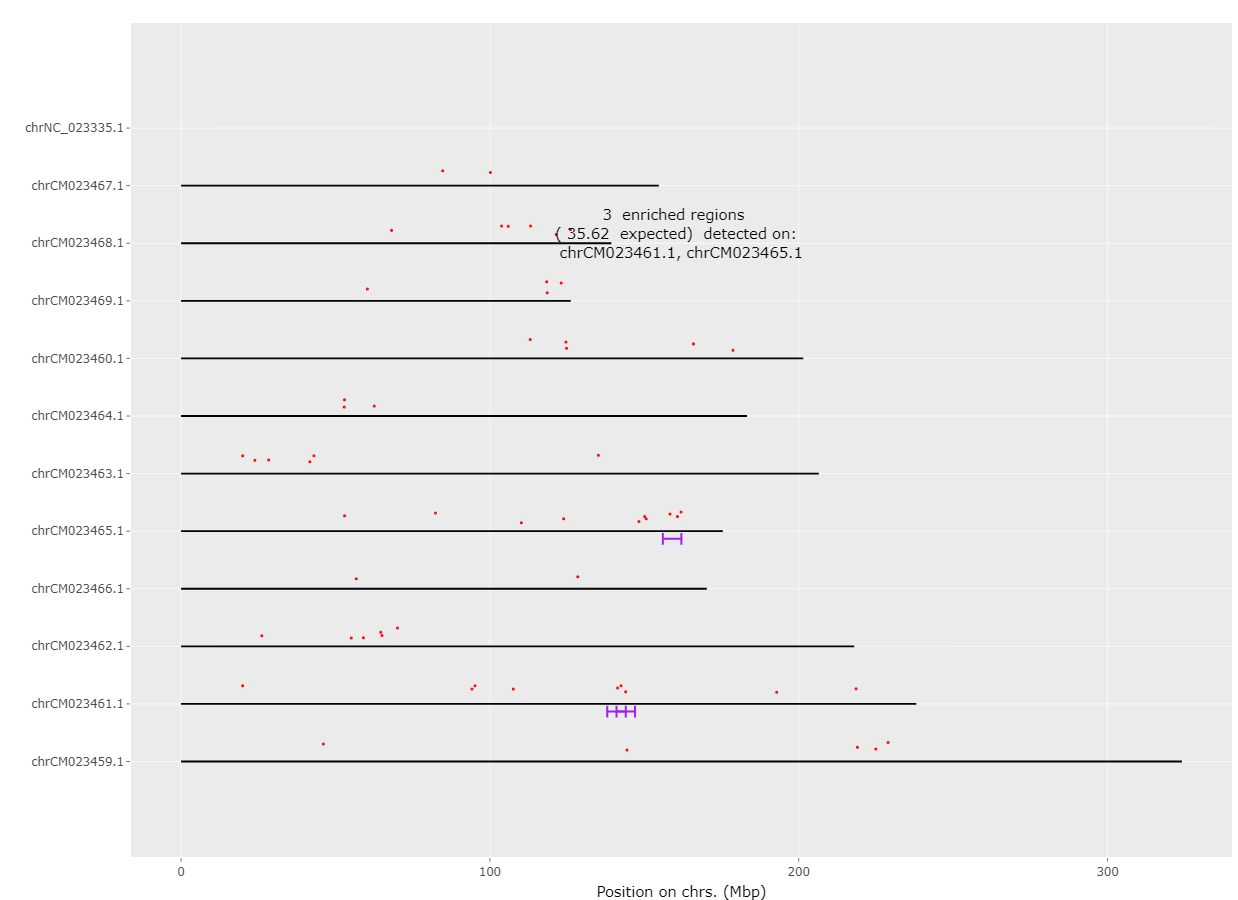


**Additional Fig. 6** Downregulated protein-coding genes restricted to *B. bovis* infection are positioned on chromosomes. Genes are represented by red dots and the purple lines indicate regions where these genes are statistically enriched, compared to the density of genes in the background. FDR<0.05.


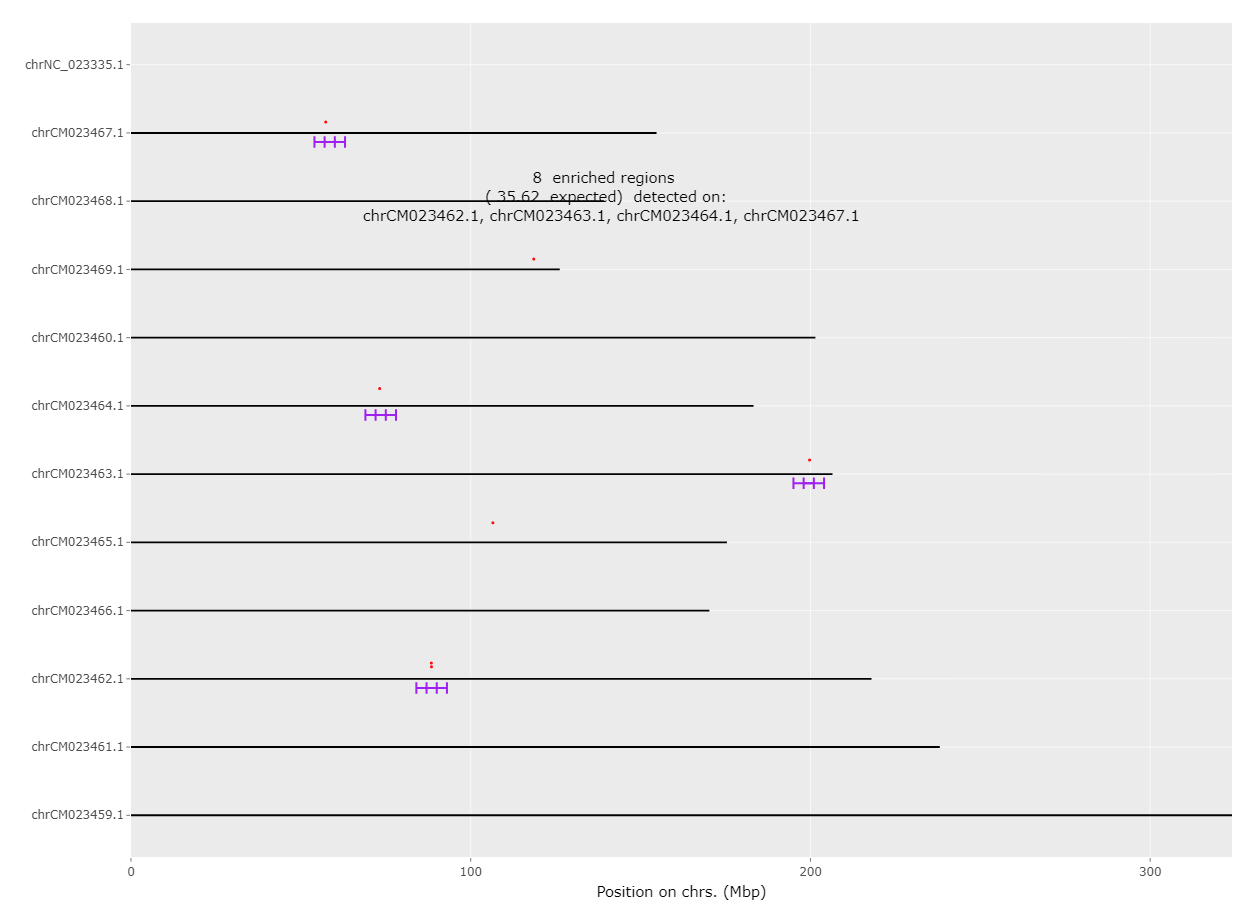


**Additional Fig. 7** Downregulated protein-coding genes restricted to *B. bigemina* infection are positioned on chromosomes. Genes are represented by red dots and the purple lines indicate regions where these genes are statistically enriched, compared to the density of genes in the background. FDR<0.05.
