# Supplementary material for: Transcriptomic analysis of Rhipicephalus microplus hemocytes from female ticks infected with Babesia bovis or Babesia bigemina
Source: Parasit Vectors. 2025 Feb 3;18:37. doi: 10.1186/s13071-025-06662-w (PMC11789329; doi:10.1186/s13071-025-06662-w)
Supplement: Supplementary file 4 — Additional File 4: Additional genes validated by qRT-PCR [file 13071_2025_6662_MOESM4_ESM.docx]

*p* ≤ 0.05

*p* ≤ 0.05

*p* ≤ 0.05

**Additional Fig. 1** Relative gene expression of upregulated hemocyte genes during *B. bigemina* infection. Keratin-associated protein 19-2-like (XM_037414510.1), Ixodidin-like (XM_037429385.1), and acanthoscurrin-2-like (XM_037413526.1) were tested. S3a: 40S ribosomal protein S3a (XM_037430639.1), Rpl4: ribosomal protein L4 (CV447629.1), and Gapdh: glyceraldehyde-3-phosphate dehydrogenase (CK180824) were utilized for qRT-PCR normalization.
